# Trophic analysis of the fish community in the Ciénega Churince, Cuatro Ciénegas, Coahuila

**DOI:** 10.7717/peerj.3637

**Published:** 2017-09-04

**Authors:** Ariana Hernández, Hector S. Espinosa-Pérez, Valeria Souza

**Affiliations:** 1 Departamento de Zoología, Instituto de Biología, Universidad Nacional Autónoma de México, Ciudad de México, México; 2 Departamento de Ecología Evolutiva, Instituto de Ecología, Universidad Nacional Autónoma de México, Ciudad de México, México

**Keywords:** Churince, Trophic analysis, Fish community, Stomach contents, Index of relative importance

## Abstract

Fish diets were analyzed to evaluate the dynamic trophs of the fish community in the Churince wetland system of the Cuatro Ciénegas, where the fauna consists of nine species: endemic, native and introduced. In nine sampling events (between February 2011 and May 2014) 556 specimens of all nine species were collected. Stomach contents were analyzed and the Relative Importance Index (IRI) was calculated. The feed coefficient (Q) of the diets and the accumulated trophic diversity (Hk), as well as the amplitude of the trophic niche were evaluated. Feeding strategies in the fish community were found to be eurifagic. The main foods in general were insects, crustaceans, gastropods, plants and teleosts. According to the average linkage method, four functional trophic groups were defined, with no higher consumption species; nevertheless all were regulators, mainly invertebrates. Therefore, the chain reaction in food control was higher from top to bottom, meaning a downwards dietary control.

## Introduction

The Chihuahuan desert is the largest in North America and the second most diverse in the world. It is a shared territory between the United States and Mexico. The largest eco-region in Mexico (WWF, 2012). The Cuatro Ciénegas basin is located in Coahuila. It has a great variety of singular aquatic environments formed by complex systems of subterranean currents, swamp-like wetlands, springs, canals, rivers, lakes, marshes (ciénegas) and temporal pools (Souza et al., 2004). The system provides refuge for different taxonomic groups, mainly crustaceans, mollusks, fish, birds, mammals, insect larvae, mites and terrestrial and aquatic plants (Carabias et al., 1999). The 50% of the fish species found in the wetland are endemic (Espinosa-Pérez, 1993), which led to its designation as a Biosphere Reserve (Reserva de la Biósfera) in 1994 (Instituto Nacional de Ecologia, 1999; Wolaver et al., 2007). There are seven major hydrographic systems in the basin: Becerra, Río Mezquites, Río Puente Chiquito, Tío Cándido, Santa Tecla, Río Salado de los Nadadores and Churince (Minckley, 1969). The latter is nowadays the least altered by human activity (Zamudio-Valdés, 1991).

The aquatic environments in Cuatro Ciénegas have been altered mostly because of grazing activities, agricultural expansion and the ceding of water rights (WWF, 2012). Native and endemic fish fauna in the Churince system was endangered by the introduction, in 1996, of the exotic Jewel Fish *Hemichromis guttatus* Günther, 1862 (Contreras-Balderas & Ludlow, 2003).

Feeding ecology and relationships of the species in a fish community may be evaluated through the analysis of stomach contents. This is the best known and most used method for the study of fish diets and can also be useful in the development of strategies for the sustained management of aquatic ecosystems (Valente, 1992; Amundsen, Gabler & Staldvik, 1996; Segatti & Luciana, 2003).

This study examined the fish community with the methods described by Graham & Vrijenhoek (1988). Our objective was to describe the trophic dynamics and relationships of the community, considering that food composition the most important factor in the conformation of the ecological niche (Krebs, 1989). The roles that fish species play in trophic chains are determined by the volume, quantity and presence of food items. These variables are considered approximations to the predator feeding strategies (Hyslop, 1980; Valente, 1992; Segatti & Luciana, 2003) and were applied to the Index of Relative Importance (IRI) proposed by Pinkas, Oliphant & Iverson (1971). The IRI annuls possible bias in individual variables, provides a more precise description of the diet importance and facilitates comparisons among studies (Bigg & Perez, 1985; Cortés, 1997).

## Materials and Methods

### Study area

The Cuatro Ciénegas basin is an inter-mountain closed basin. The climate is arid, with an average mean rainfall of less than 200 mm.; summer day temperatures occasionally above 44 °C and winter temperatures below 0 °C (Minckley, 1969). Despite the dry climate, the basin harbors an extensive system of springs, streams and pools.

The main source of the subterranean water is old water deposited by the end of the late-Quaternary (Meyer, 1973; Wolaver et al., 2007), which is low in levels of NaCl and carbonates, and rich in sulfates, magnesium and calcium. One of the most important characteristics of the ecosystem is its low level of phosphorus, both in the water and in the soil, in comparison to other similar environments (Elser et al., 2005). Although phosphorus is an essential nutrient in various cellular processes and is not an abundant element in the world, Cuatro Ciénegas’ phosphorus levels are below the detection level (0.3 M) (Minckley, 1969; Scanlan, Mann & Carr, 1993).

The Cuatro Ciénegas basin’s water systems are probably connected in a natural way, either underground or over the surface during the rainy season, although man-made irrigation channels have modified or interconnected most of the systems, therefore eliminating many habitats by diminishing the level of the underground water (Minckley & Cole, 1968).

**Figure 1:**
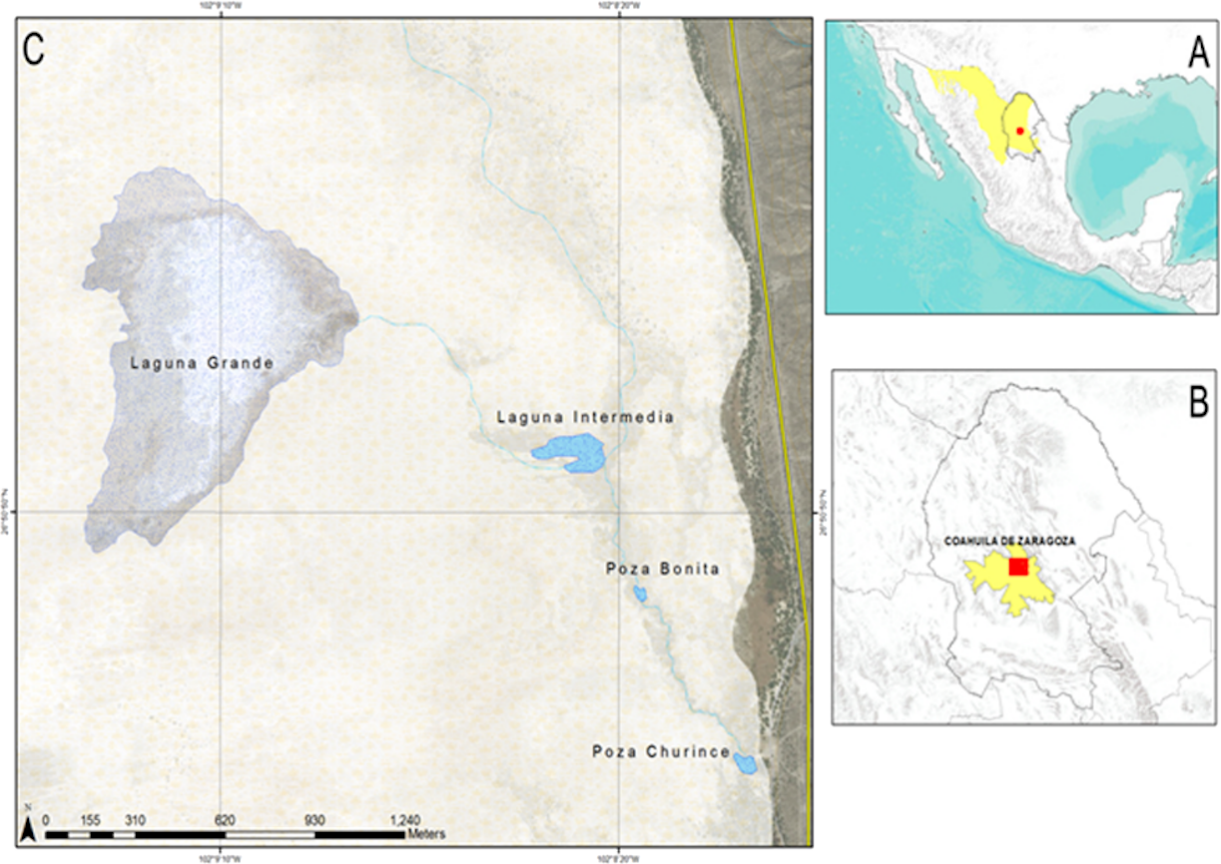
Map of Cuatro cienegas location. (A) Mexico and the Chihuahuan desert, (B) Coahuila and Cuatro Ciénegas and (C) the Ciénega Churince.

The present study was done in the Churince aquatic system, which was divided into five collecting areas, considering the water level and its physico-chemical characteristics: (1) Laguna Grande o Laguna Churince, (2) Laguna Intermedia, (3) Poza Bonita, (4) Rio Churince and (5) Poza Churince (Fig. 1) (Zamudio-Valdés, 1991; Hendrickson et al., 2008). The Churince aquatic system is the oldest marsh in the Cuatro Ciénegas valley and was considered the least altered by human activity (Zamudio-Valdés, 1991). However from 2006 to 2009 a sudden 30 m decrease in the water level table was recorded. This decrease affected mainly the Laguna Grande, turning it into a desolate space full of saltpeter (Carrera, 2011).

### Fish sampling

Nine samplings were carried out in the Ciénega Churince from February 2011 to May 2013 with the collection permit SAGPA/DGVS/02138, and PPF/DGOPA-140/14. Specimens were collected with a cast net of 3 × 1.5 m, 0.5 cm mesh size seine, and with dip-nets and minnow traps for jewel fish. Fish were preserved, analyzed and deposited in the Colección Nacional de Peces of the Instituto de Biología, UNAM.

### Stomach content analysis

Fish stomachs were Hyslop (1980) and Laevastus (1980). Prey were documented for volumetric percentage: food volume with respect to stomach content volume; numerical percentage: the number of individuals of each food category out of the total number of individuals of all food categories in the stomach; and frequency of occurrence expressed as a percentage: the number of stomachs with one to more individuals of each food category out of all the analyzed stomachs. Data was integrated into the Index of Relative Importance (IRI) (Pinkas, Oliphant & Iverson, 1971), and the feeding coefficient, which determines the importance of food components. For the feeding coefficient, *Q* > 200 is considered as preferred prey, *Q* ≥ 20 as secondary prey and *Q* < 20 as rare prey. Jaccard coefficient was used to calculate the similarity of taxa ingested among fish species, in addition to estimating the overlap by means of the Piankas index. The accumulated trophic diversity (Hk) was calculated according to the method applied by Olguín, Attamedo & Beltzer (2012). Similarity of diets with respect to the identified taxa was also measured with the Jaccard index (Villareal et al., 2006)

*Trophic level* or position was determined in accordance to the feeding habits type of each species with respect to the Index of Relative Importance and the feeding coefficient. Trophic positions were defined as 1st level (primary producers), 2nd level (primary, secondary and tertiary) and 3rd level (decomposers) categories (Tyler, 1994).

*Trophic niche amplitude* quantitatively defines an organism as a generalist when it feeds on a variety of food items, and as a specialist when it preferentially eats one type of prey. The standardized Levins index was used to estimate the niche amplitude from the uniformity in the distribution of the individuals among the diverse food resources. Values of 0 to 0.60 characterize an organism as a specialist and values above 0.60 as a generalist (Krebs, 1989).

### Trophic chain

The degree of similarity of the fish diets was determined using accumulative hierarchical and numerical classification analysis techniques, in order to define groups of species that share similar prey. The establishment of similar trophic groups was based on the average linkage method. A matrix was constructed using the Bray-Curtis dissimilarity index. Dendrograms were used for graphic representations.

## Results

### Collected specimens

**Figure 2:**
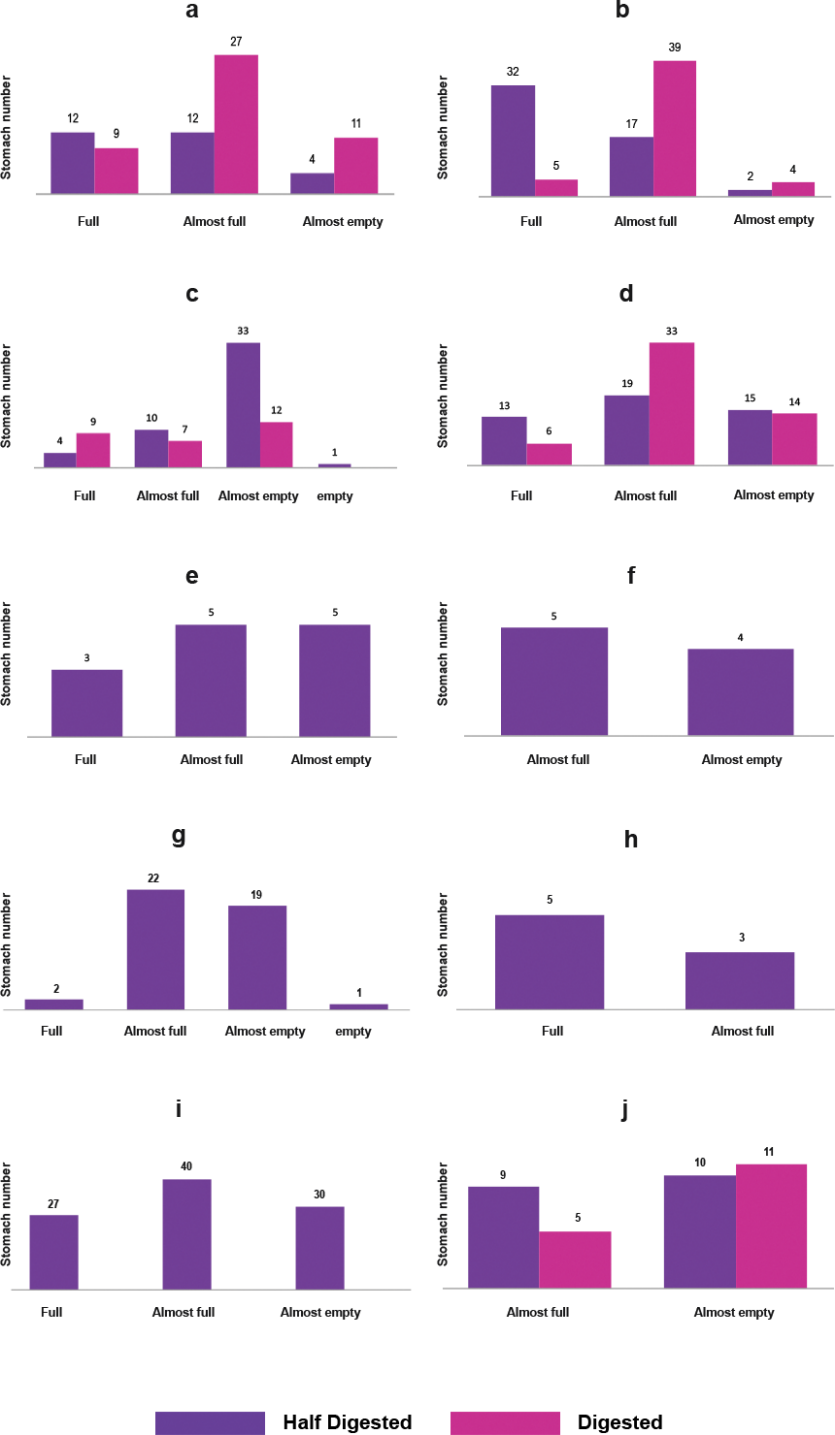
Qualitative description of the fish stomachs: (A) *C. atrorus,* (B) *C. bifasciatus,* (C) *G. marshi* G1, (D) *G. marshi* G2, (E) *L. macrochirus,* (F) *L. megalotis,* (G) *M. salmoides,* (H) *H. minckleyi,* (I) *H. guttatus,* (J) *C. xanthicara*.

A total of 569 specimens of nine species and five families of fish were collected (Table 1). As it was not possible to identify the stomach contents of all the groups down to the lowest possible taxonomic level, these were characterized as general categories, allowing the results to be standardized, however, they are listed in annex 1 for consultation. The main qualitative characteristics of the stomach contents of the community are shown in Fig. 2, the food description of each species are as follows:

**Table 1:** Collected species, number of food categories and the Levins index.

Species	NOM-059	Number of specimens analyzed	Number of food categories	Levins index
*C. atrorus*	E, A	75	6	0.77
*C. bifasciatus*	E, A	99	8	0.75
*G. marshi* G1	N, A	76	7	0.86
*G. marshi* G2	N, A	100	6	0.95
*L. macrochirus*	N	13	6	0.60
*L. megalotis*	N	9	7	0.75
*M. salmoides*	N	47	5	0.95
*H. minckleyi*	E, P	17	8	0.77
*H. guttatus*	Ex	102	7	0.34
*C. xanthicara*	E, P	35	6	0.56

**Notes.**
Classification of species in NOM-059-SEMARNAT-2010 N native E endemic Ex exotic A threatened P endangered

**Figure 3:**
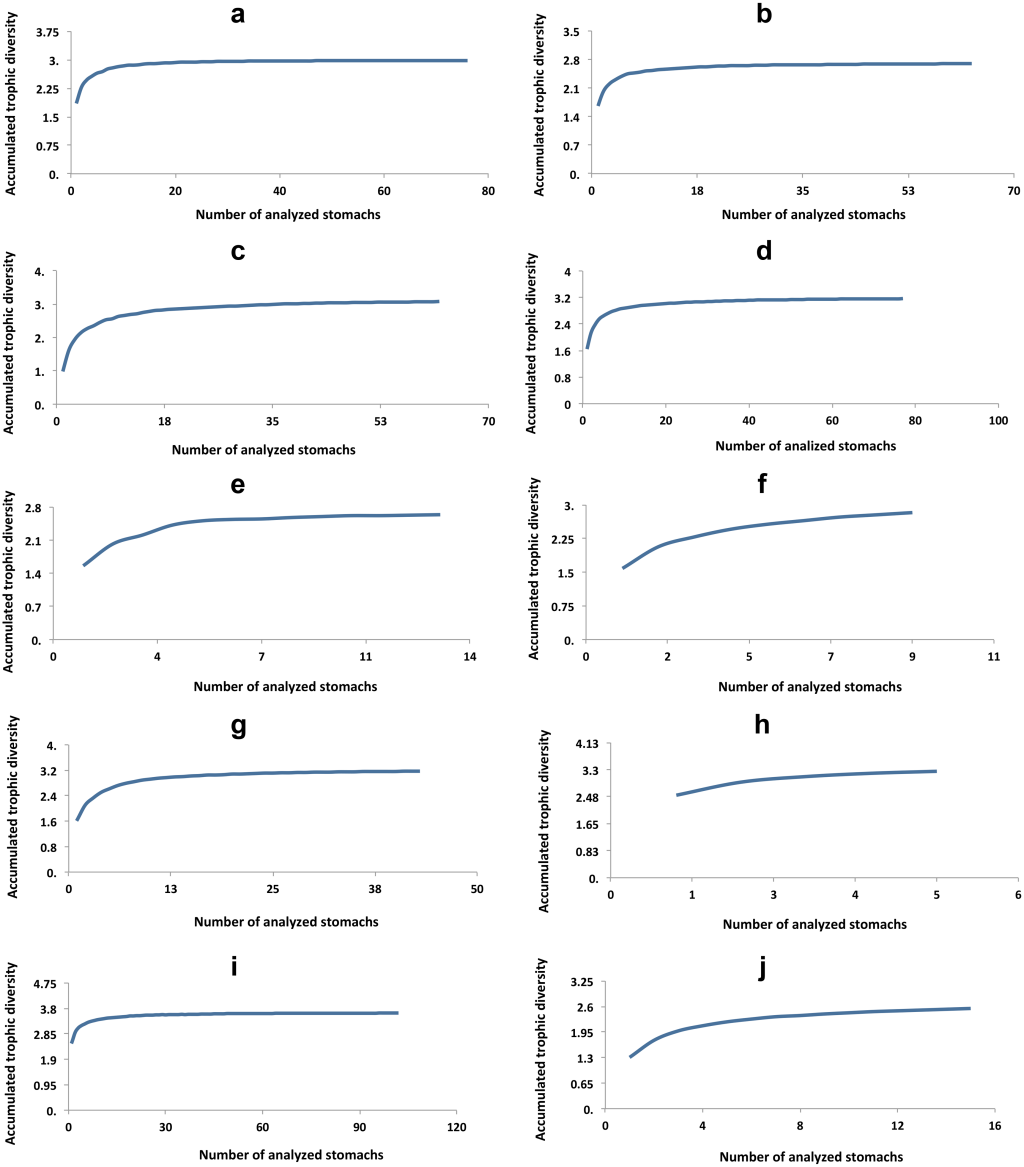
Accumulated trophic diversity (Hk) of the fish community: (A) *C. atrorus,* (B) *C. bifasciatus,* (C) *G. marshi* G1, (D) *G. marshi* G2***,*** (E) *L. macrochirus,* (F) *L. megalotis,* (G) *M. salmoides,* (H) *H. minckleyi,* (I) *H. guttatus,* (J) *C. xanthicara*.

**Figure 4:**
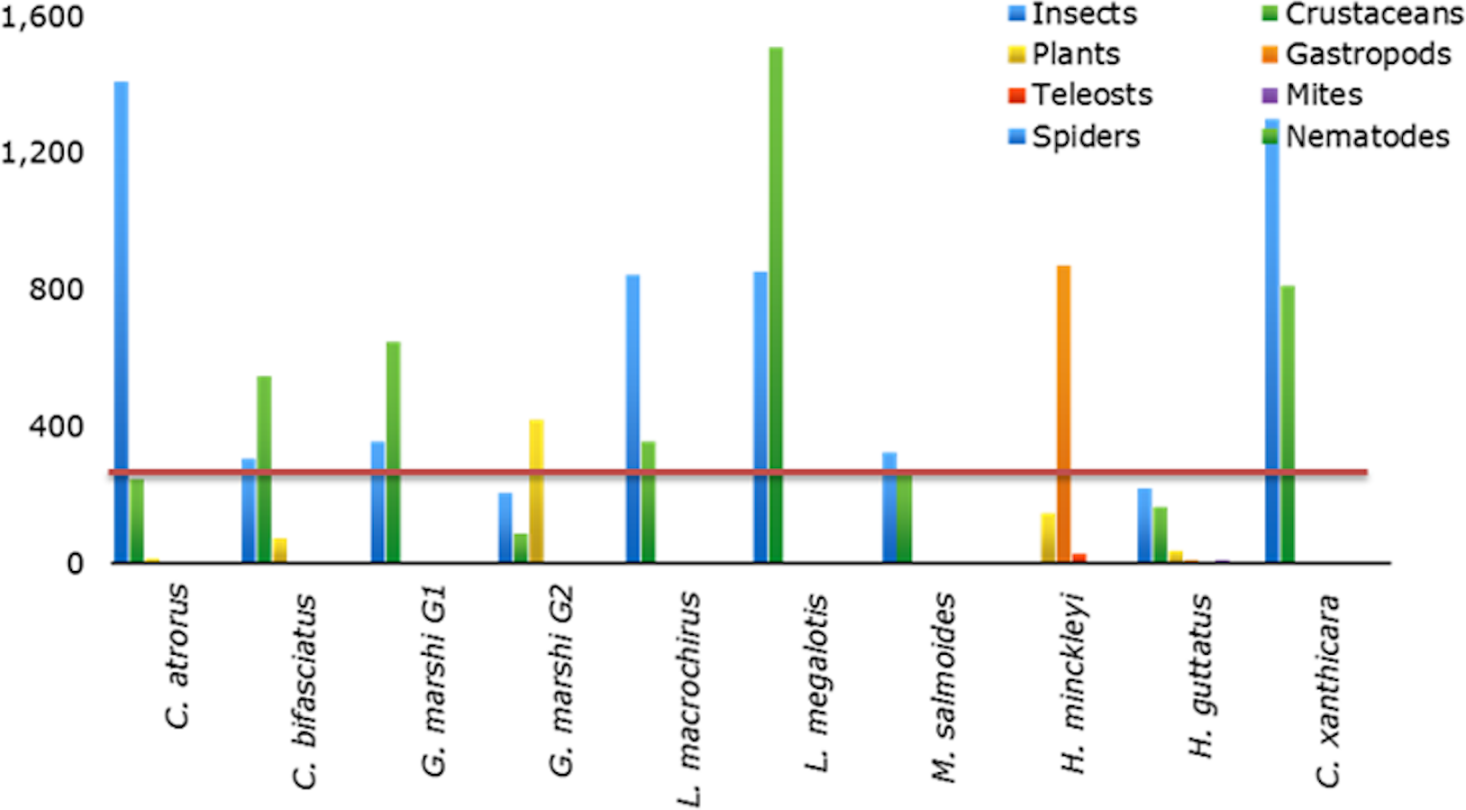
Coefficient of fish feed that delimits the categories of preferential foods in: primary, secondary and rare.

*Cyprinodon atrorus* Miller, 1968, cachorrito del Bolsón –Bolsón Pupfish. In the Churince aquatic system this pupfish is distributed from Laguna Intermedia to half way along the Río Churince. This species is considered threatened (NOM-59-SEMARNAT-2010). 75 specimens were analyzed (Table 1). The accumulated trophic diversity (Hk) was 3, reaching the asymptote of the curve (Fig. 3). The amplitude of the trophic niche showed a value of 0.77 (Table 1). There were 27 species as stomach items, represented in six categories. The contribution of each food category, according to the index of relative importance, was: insects 77.3, crustaceans 17.9, plants 3.25, teleosts 1.46, mites 0.18, and gastropods 0.05 (Table 2). According to the dietary coefficient, the preferred categories were crustaceans and insects (Fig. 4).

*Cyprinodon bifasciatus* Miller, 1968, cachorrito de Cuatro Ciénegas—Cuatro Ciénegas Pupfish. This species is restricted to thermal waters (26.7 °C–34.5 °C) in or near springs, streams and wetlands that have a constant flow of hot water and unchanging physical characteristics (Arnold, 1972). It is considered a threatened species (NOM-59-SEMARNAT-2010). 99 stomachs were reviewed (Table 1), in which 27 food items were found, within eight categories. The contribution of each food category according to IRI was: 55.18 crustaceans, 29.14 insects, 11.71 plants, 2.83 teleosts, 0.72 mites, 0.22 gastropods, 0.11 nematodes, and 0.1 spiders (Table 2). The accumulated trophic diversity (Hk) was 2.6, reaching the asymptote of the curve (Fig. 3). This species presented a value of 0.75 in the amplitude of its niche (Table 1). The feed ratio indicates that the categories of insects and crustaceans are the main food and plants are the secondary (Fig. 4).

**Figure 5:**
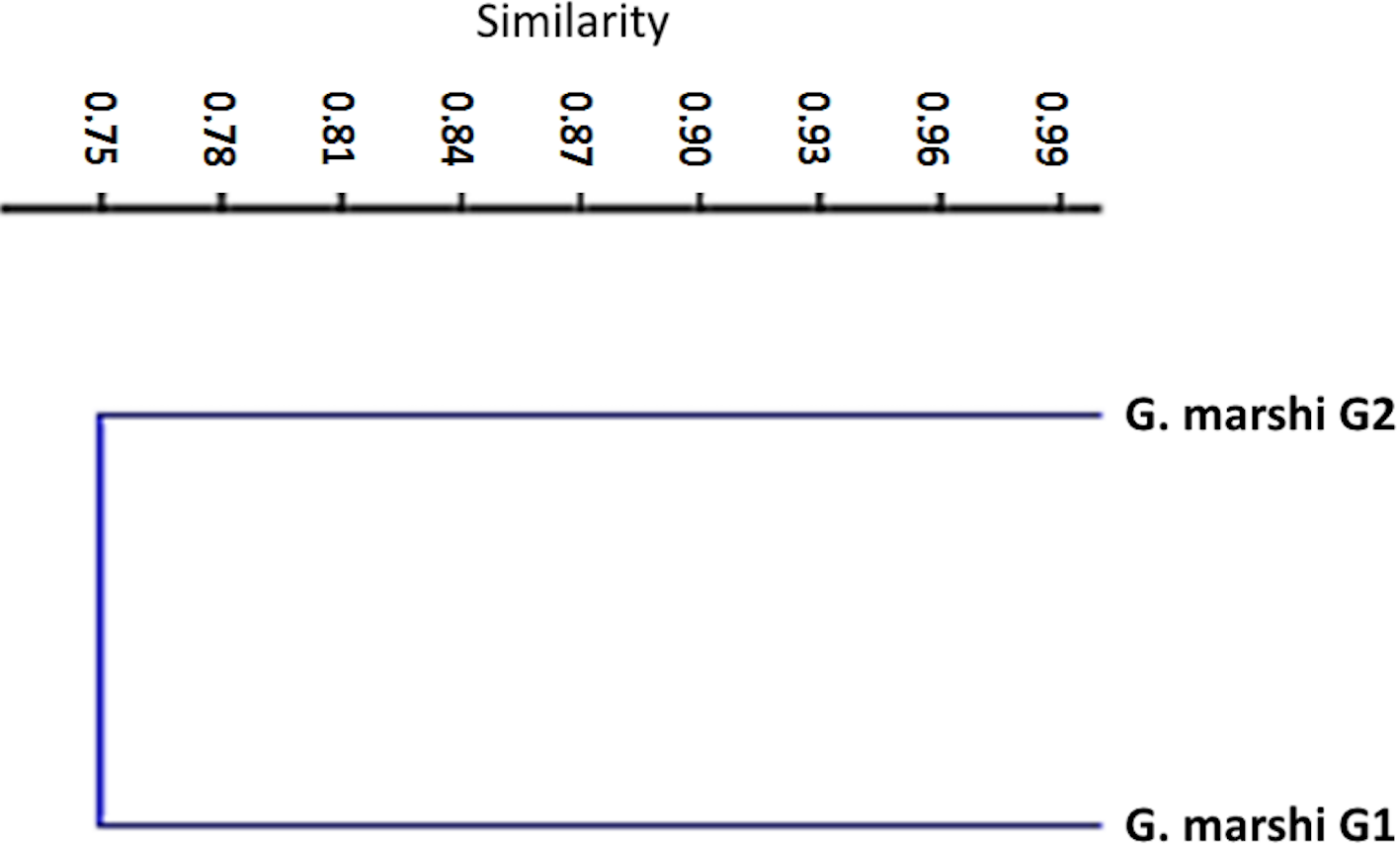
Jaccard dendrogram for measure similitude between the two groups of *G. marshi*.

*Gambusia marshi* Minckley & Craddock, 1962, guayacón de los Nadadores—Robust Gambusia. This species swims near the surface, it’s aggressive and distributed widely in the Bolsón de Cuatro Ciénegas (Miller, Minckley & Norris, 2009). The species is considered threatened (NOM-59-SEMARNAT-2010). It is the only species found throughout the whole Churince aquatic system. In order to analyze the diet, the collected specimens were divided into two groups, according to the physical and chemical characteristics of the system (e.g., temperature, plants in and outside the water, and species with which they interact in the marsh). The first group (70 specimens) was distributed from Laguna Intermedia to the middle of the Rio Churince (Table 1), the same as *C. atrorus*; while the second group (106 specimens) is distributed from the middle of the Rio Churince to Poza Bonita, the same as *C. bifasciatus*. In the first group, 32 food items in 8 categories were found. According to the IRI, they contribute as follows: insects 48.32, crustaceans 43.86, teleosts 3.98, mites 1.44, spiders 1.11, plants 0.75, nematodes 0.35, and gastropods 0.04. The feeding coefficient indicates that insect and crustacean categories are preferred (Fig. 4). The accumulated traffic diversity (Hk) yielded a value of 3, reaching the asymptote of the curve (Fig. 3). Its niche amplitude has a value of 0.86 (Table 1). In the second group were found 44 species in six categories, which according to IRI, contributed as follows: plants 39.22, insects 35.95, crustaceans 22.62, teleosts 1.00, mites 1.22, and spiders 0.67. The traffic diversity (Hk) is 2.5, reaches the asymptote of the curve (Fig. 3). The amplitude of the traffic niche is 0.95 (Table 1). The feed ratio determines that the plant and insect categories are preferential, and the crustaceans are secondary items in their diet. Jaccard’s dendrogram of both groups’ diets expressed 75% of similarity (Fig. 5).

**Table 2:** Summary of the trophic spectrum of the Ciénega Churince’s fish community, through index of relative importance.

Genus species	Crustaceans	Mites	Spiders	Insects	Gastropods	Teleosts	Nematodes	Plants
*C. atrorus*	17.49	0.18	0.00	77.53	0.05	1.46	0.00	3.25
*C. bifasciatus*	55.18	0.72	0.10	28.94	0.22	2.83	0.11	11.71
*G. marshi G1*	43.86	1.44	1.11	48.32	0.04	3.98	0.35	0.75
*G. marshi G2*	22.47	1.21	0.67	35.71	0.00	0.99	0.00	38.96
*L. macrochirus*	29.82	0.23	0.00	67.40	0.00	1.33	1.09	0.13
*L megalotis*	63.05	0.00	0.13	34.02	0.82	0.58	0.31	0.30
*M. salmoides*	40.12	2.14	0.00	56.14	0.00	0.82	0.00	0.27
*H. minckleyi*	4.82	0.90	0.13	7.35	54.46	7.21	0.52	24.74
*H. guttatus*	35.04	4.88	0.02	43.31	4.48	2.12	0.01	9.98
*C. xanthicara*	45.90	0.26	0.00	53.63	0.04	0.03	0.00	0.05

*Lepomis macrochirus* Rafinesque, 1819, mojarra oreja azul—Bluegill Sunfish. This species is a bottom dweller in temperate slow-moving waters and deep, calm, stagnant areas where aquatic plants and other types of vegetation are common (Miller, Minckley & Norris, 2009). 13 specimens were analyzed, of which 18 food items were identified within 6 categories. Their contribution according to IRI was: 67.4 insects, 29.82 crustaceans, 1.33 teleosts, 1.09 nematodes, 0.13 plants, and 0.23 mites (Table 2). The feed ratio expressed that insects and crustaceous are the preferred food categories (Fig. 4). The accumulated trophic diversity is 2.8, and did not reach the asymptote of the curve (Fig. 4). The amplitude of the trophic niche has a value of 0.60 (Table 1).

*Lepomis megalotis* (Rafinesque, 1820), orejona roja—Longer Sunfish. This species is usually found in the higher parts of rivers and their tributaries, in standing clear or muddy waters, and in shallow water bodies. Twelve stomachs were reviewed in which 24 food items of 7 categories were found (Table 1). According to the IRI, the food categories contribute as follows: 63.05 crustaceans, 34.92 insects, 0.82 gastropods, 0.59 teleosts, 0.31 nematodes, 0.3 plants, and 0.13 spiders (Table 2). The feed ratio classifies crustaceans and insects as the main items. The accumulated trophic diversity reaches 3 without reaching the asymptote of the curve (Fig. 4). The amplitude of the trophic niche has a value of 0.75 (Table 1).

*Micropterus salmoides* (Lacepède, 1802), lobina negra—Largemouth Bass. This is a benthic species that tolerates a wide variety of conditions, but prefers warm, moderately clear, slow-moving or standing waters. 48 stomachs were analyzed in which 41 food items were identified and classified into five categories (Table 1). According to IRI, they contribute to the diet as follow: 56.14 insects, 40.12 crustaceans, 2.14 mites, 0.82 teleosts, 0.27 plants (Table 2). The feed coefficient classifies the crustaceans and insects as primary categories in their feeding (Fig. 4). The accumulated trophic diversity has a value of 3.2, reaching the asymptote of the curve (Fig. 3). The amplitude of the traffic niche is 0.95 (Table 1).

*Herichthys minckleyi* (Kornfield & Taylor, 1983), mojarra de Minckleyi—Minckley’s Cichlid. Species classified as endangered (NOM-59-SEMARNAT-2010). Minckley’s Cichlid has three morphological types, or “morphs” (Kornfield & Taylor, 1983; Minckley, 1969; Husley, Hendrickson & García de León, 2005). The main polymorphism is in the pharyngeal teeth: It is possible to find individuals with very big and strong teeth (molariform), as well as very fine, pointed and delicate teeth (papilliform). It has been documented that individuals with molariform teeth eat mainly snails, while those with papilliform teeth feed on organic matter, among other things (Minckley, 1969; Kornfield & Taylor, 1983). The piscivorous morph has more elongated head and body and its diet includes especially *C. bifasciatus* (Minckley, 1969; Kornfield & Taylor, 1983). Nine specimens were collected, in which 28 food items were identified and classified into eight categories. According to IRI, items contribute to the diet as follow: gastropods 54.46, plants 24.74, crustaceous 4.82, spiders 0.13, insects 7.35, teleosts 7.21, mites 0.9, and nematodes 0.52 (Table 2). The feed factor classifies gastropods as the primary category and plants and teleosts as secondary food (Fig. 4). The amplitude of the trophic niche is 0.77 (Table 1). The accumulated trophic diversity reaches 3.3 without reaching the asymptote of the curve (Fig. 3).

*Hemichromis guttatus* Günther, 1862, jewel fish—Jewel Cichlid. This opportunist feeder lives in the water column. Fish of this genus are bi-parental; they build nests on the bottom and care for them together. The species is native to Africa and exotic to the area (NOM-59-SEMARNAT-2010). It was first reported in March 1996 in Poza Churince (Contreras-Balderas & Ludlow, 2003). It is distributed in Poza Bonita, Río Churince and Poza Churince. A total of 102 stomachs were analyzed, in which 77 food items of seven categories were identified (Table 1). According to IRI, items contribute to the diet as follow: 43.31 insects, 35.04 crustaceous, 9.98 plants, 4.88 mites, 4.48 gastropods, 2.12 teleosts, 0.02 spiders, and 0.01 nematodes. The feed coefficient classifies insects as the primary food category and the crustaceans and plants as secondary (Fig. 4).

*Cyprinella xanthicara* (Minckley & Lytle, 1969), sardinita de Cuatrociénegas—Cuatrocienegas Shiner. Species classified as endangered (NOM-59-SEMARNAT-2010). This pelagic fish lives in large springs of clear water, in streams fed by springs, in areas with currents and in where there is contact between currents and standing water. It tends to concentrate above and below riffles over marl, gravel, rocks and flocculated clay. It is more abundant in the high parts of streams fed by springs, just below the source of the water (Miller, Minckley & Norris, 2009). In the Churince system, it is distributed only in Laguna Intermedia. Thirty - five stomachs were analyzed, in which 19 food items of six food categories were identified. According to IRI, items contribute to the diet as follow: 53.63 insects, 45.9 crustaceans, 0.26 mites, 0.05 plants, 0.04 gastropods, and 0.03 teleosts. The feed coefficient classifies crustaceans and insects as the main categories in the diet of this species. The accumulated trophic diversity is 2.6 without reaching the asymptote of the curve. The amplitude of the trophic niche was estimated at 0.56.*Overlapping diets.* The Pianka index, bold as a result; Observed Index: 0.55, Lower-tail *P* > 0.999 and Upper-tail *P* < 0.001.

**Figure 6:**
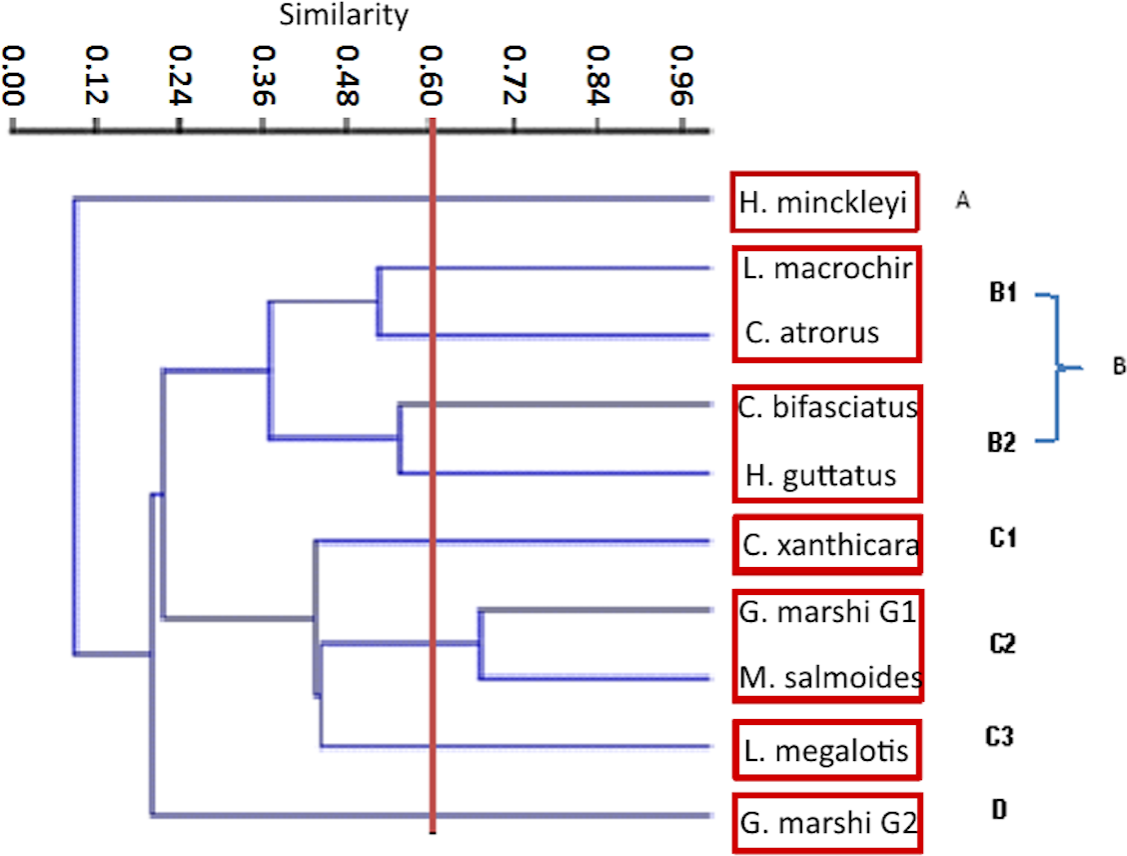
Jaccard cluster species against food items. The Pianka index, and threw as a result 0.49.

## Discussion

The sample size of each species was different due to various factors such as the season, but it mainly reflected the distribution of each species in the aquatic system. Water level, physico-chemical characteristics, exposure to evaporation, and vegetation, among other factors vary throughout the year; and each species responds to the changes in different ways (Table 1). The useful stomachs for this study were those half and totally full, with half-digested and fresh content (Fig. 2).

Arnold (1972) and Minckley (1969) stated that the diet of *C. atrorus* is herbivorous, considering the elongated and convoluted form of the intestine. However in this study *C. atrorus* was found a secondary predator, feeding mainly on insects and crustaceans, with a generalist feeding habit, and large trophic niche. Minckley (1969) and Miller, Minckley & Norris (2009) analyzed the stomach contents of adult specimens of *C. bifasciatus* and found organic matter and substrate residues. They thus stated that the latter species fed on plants, animals and even on its own eggs. In this study, however, insects and crustaceans were recorded as the preferred categories, with plants as the secondary food item. We consider this species as an omnivore, which allows it to feed at different trophic levels (as a primary or secondary consumer).

The first group of *G. marshi* fed mainly on insects and crustaceans, with a greater importance on the number and volume than on the occurrence. According to the Levins index, its diet was generalized and heterogeneous with an omnivore feeding habit, which allows it to be in different trophic levels. The diet of the second group of *G. marshi* included plants and insects as the dominant, as well as the preferred food items, with crustaceans as the secondary food item. As in the first group, the Levins index established its diet as generalized and heterogeneous with omnivore habits. The Jaccard’s similarity index dendrogram (Fig. 6) expressed 75% of similarity in the diet of both groups, indicating that they have similar feeding behavior, although the feeding categories recorded different importance in each group. Miller, Minckley & Norris (2009) stated detritus, insects and other invertebrates as the main food items of *G. marshi,* as well as the results of this study, that established the main feeding importance of insects, crustaceans and plants. Both *Gambusia* groups are considered as generalists and omnivores.

Stuber, Gebhart & Maughan (1982) classified *L. macrochirus* as an opportunist consumer, as it was able to change its diet in accordance to the availability of food items, though it fed mainly on zooplankton and small insects. In this study, we documented insects and crustaceans as the dominant feeding categories of this species, with a predominance of heavy and numerous prey items. The diet of this species is thus generalized and heterogeneous.

Miller, Minckley & Norris (2009) characterized *L. megalotis* as an opportunist that fed on easy-to-catch prey, such as insects and small invertebrates. In this study, crustaceans and insects were recorded as the first and second most important feeding categories; this species’ diet was therefore defined as generalized and heterogeneous.

Miller, Minckley & Norris (2009) stated that *M. salmoides* juveniles fed on small crustaceans, insects and insect larvae, while the adults ate mainly fish, prawn, large insects and occasionally frogs, mouses or other animal that swam or fell into the water. Juveniles and adults were not compared in this study but, in general, insects were recorded as the dominant food item of the species, with crustaceans also as a preferred food and with a greater contribution in number and total weight of prey. This species is hence classified as a secondary consumer with heterogeneous and generalized feeding habits.

In this study, specimens of *Herichthys minckleyi* were found feeding mainly on gastropods, with teleosts and plants as secondary food items. This indicates that only one morph was sampled. The species is considered a generalist with a heterogeneous diet.

Jewel fish are known to be an opportunistic species. In this study, specimens were found feeding mainly on insects, with plants and crustaceans as secondary categories. The Levins index classified this species as an omnivore with a specialized diet.

Page & Burr (1991) reported that *C. xanthicara* ate mainly algae and other plants. However, this study found crustaceans and insects as the preferred categories in its diet. This species has a very characteristic stomach, as it appears to have a gizzard where digestion starts. It is defined as a species with a generalized and heterogeneous diet.

**Figure 7:**
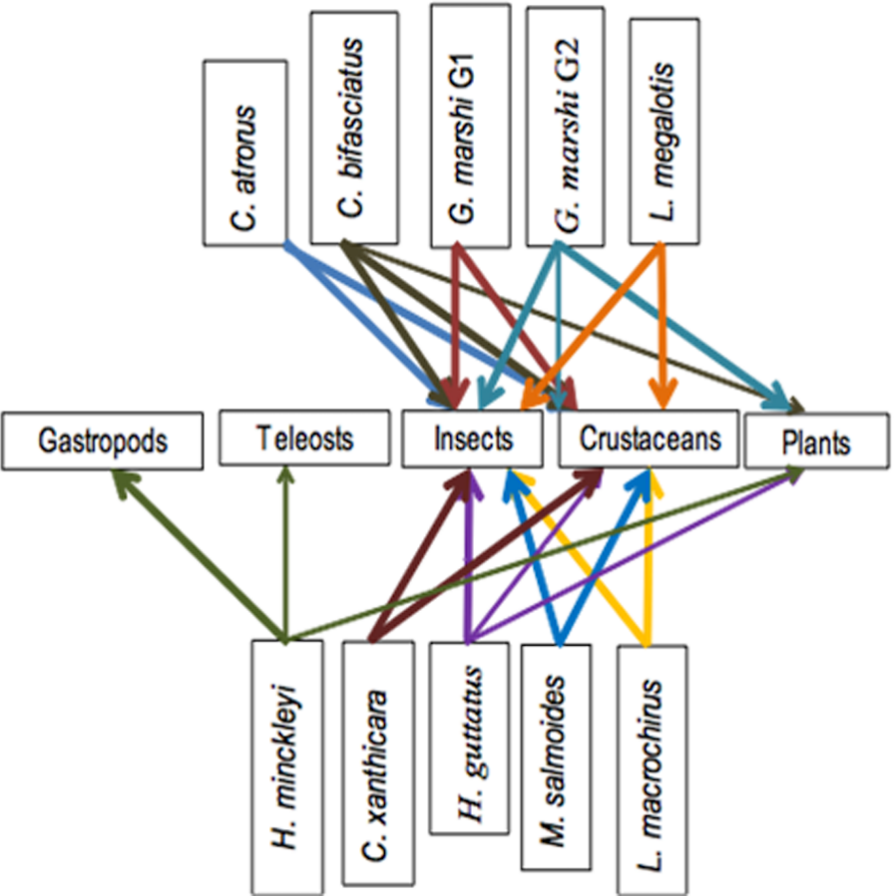
Simplified trophic chain.

The Jaccard similarity cluster of the IRI for all the species (Fig. 7) formed four main groups: Group A with only *H. minckleyi,* which is different from all the other species. Group B, with a medium similarity among its species and therefore partitioned into the subgroups B1 with *L. macrochirus* and *C. atrorus*, and B2 with *C. bifasciatus* and *H. guttatus*. Group C with a medium similarity among its species and partitioned into the subgroups C1 with only *C. xanthicara*, C2 with *G. marshi* G1 and *M. salmoides*, and C3 with *L. megalotis;* and Group D with only *G. marshi* G2. And according to the index of Pianka, which tells us that the values towards 1 are completely overlapping and towards 0 there is no overlap, we conclude that the community does not present a representative overlap.

The trophic chain of the community indicates the presence of second degree consumer species. That is carnivores and omnivores that may be found in different places within the chain. No top consumer fish species were found, as no species was strictly an ichthyophage and the ichthyophage morph of *H. minckleyi* was not collected. Even without top consumer fish species, the top-bottom chain reaction in the food control was potentially greater than the bottom-top chain reaction, as the fish community regulates the populations of plants and aquatic invertebrates (Carpenter & Kitchel, 1993; Jeppesen et al., 1997; Lovgren & Persson, 2000).

The primary producers found in this study included plants and algae, though the algae were not analyzed. Souza et al. (2004) mentioned that stromatolites and photosynthetic bacteria (cyanobacteria), characteristic of the early Cambrian (540 million years ago), form the base of the food pyramid. This applies particularly to Laguna Intermedia, where stromatolites are common.

## Conclusions

The fish community of Ciénega Churince is structured by nine species within five families. The trophic spectrum of *C. atrorus*, *G. marshi* G1, *L. macrochirus*, *L. megalotis*, *M. salmoides* and *C. xanthicara* indicate that insects and crustaceans are important in their diet, as only preferred categories. The trophic spectrum of *C. bifasciatus*, *G. marshi* G2, *H. minckleyi* and *H. guttatus* differed because secondary food categories were also recorded, providing significant importance and constituting a dietary supplement. Nevertheless, items registered as parts of the fish community diet reflect only the food availability and accessibility, not the exclusive or permanent preference of the species for certain item.

The feeding of the community of fish is considered eurifagic, since its items belong to more than one preferential category. The similarity of the preferred categories has no taxonomic significance, but shows a remarkable pattern of similarity with respect to the distribution of fish species in the marsh. The trophic chain proposed in this study clustered four general functional and hierarchical groups, with no fish species of greater consumption in any group.

In conclusion, there is a low abundance of high-level fish predators, and it is estimated that their regulatory control does not fall directly on fish stocks, but it does on aquatic invertebrates and aquatic plants. Nevertheless, since variables such as the consumption of fish eggs by birds, snakes and other large organisms, as well as fisheries and other factors, were not considered or strictly analyzed, the features that may regulate fish populations could be underestimated.

##  Supplemental Information

10.7717/peerj.3637/supp-1 Supplemental Information 1 Raw data

10.7717/peerj.3637/supp-2 Supplemental Information 2 Catalog number of fish analyzed

10.7717/peerj.3637/supp-3 Supplemental Information 3 Trophic spectrum disaggregated from each species of the fish community

10.7717/peerj.3637/supp-4 Supplemental Information 4 Pianka Index

## Additional Information and Declarations

### Field Study Permissions

The following information was supplied relating to field study approvals (i.e., approving body and any reference numbers): Colección Nacional de Peces IBUNAM SAGARPA granted fishing permits no. PPF/DGOPA-140/14-15 and DGOPA00889.280211-0349 to Hector Espinosa.

### Data Availability

The following information was supplied regarding data availability: The raw data has been supplied as a Supplemental File.
