# Supplementary material for: Trophic analysis of the fish community in the Ciénega Churince, Cuatro Ciénegas, Coahuila
Source: PeerJ. 2017 Sep 4;5:e3637. doi: 10.7717/peerj.3637 (PMC5588786; doi:10.7717/peerj.3637)
Supplement: Supplemental Information 2 [file peerj-05-3637-s002.docx]

**Catalog numbers**

| Species | Catalog numbers |
| --- | --- |
| C. atrorus | CNPE-IBUNAM18857, CNPE-IBUNAM18858, CNPE-IBUNAM18859, CNPE-IBUNAM18860, CNPE-IBUNAM18868, CNPE-IBUNAM18883, CNPE-IBUNAM18892, CNPE-IBUNAM18900, CNPE-IBUNAM18913, CNPE-IBUNAM18926, CNPE-IBUNAM18933, CNPE-IBUNAM18937, CNPE-IBUNAM18943, CNPE-IBUNAM18957, CNPE-IBUNAM18958. |
| C. bifasciatus | CNPE-IBUNAM18870, CNPE-IBUNAM18876, CNPE-IBUNAM18889, CNPE-IBUNAM18897, CNPE-IBUNAM18898, CNPE-IBUNAM18903, CNPE-IBUNAM18905, CNPE-IBUNAM18916, CNPE-IBUNAM18928, CNPE-IBUNAM18939, CNPE-IBUNAM18940, CNPE-IBUNAM18947, CNPE-IBUNAM18948 |
| G. marshi | CNPE-IBUNAM18856, CNPE-IBUNAM18861, CNPE-IBUNAM18865, CNPE-IBUNAM18872, CNPE-IBUNAM18874, CNPE-IBUNAM18878, CNPE-IBUNAM18879, CNPE-IBUNAM18884, CNPE-IBUNAM18887, CNPE-IBUNAM18891, CNPE-IBUNAM18893, CNPE-IBUNAM18896, CNPE-IBUNAM18899, CNPE-IBUNAM18901, CNPE-IBUNAM18906, CNPE-IBUNAM18909, CNPE-IBUNAM18912, CNPE-IBUNAM18915, CNPE-IBUNAM18920, CNPE-IBUNAM18923, CNPE-IBUNAM18925, CNPE-IBUNAM18929, CNPE-IBUNAM18931, CNPE-IBUNAM18932, CNPE-IBUNAM18934, CNPE-IBUNAM18936, CNPE-IBUNAM18938, CNPE-IBUNAM18942, CNPE-IBUNAM18944, CNPE-IBUNAM18946, CNPE-IBUNAM18949, CNPE-IBUNAM18953, CNPE-IBUNAM18960. |
| L. macrochirus | CNPE-IBUNAM18864, CNPE-IBUNAM18895, CNPE-IBUNAM18921, CNPE-IBUNAM18927. |
| L. megalotis | The specimens used for the analysis were not deposited in the coloection due to deterioration, which they presented after using them. |
| M. salmoides | CNPE-IBUNAM18886, CNPE-IBUNAM18888, CNPE-IBUNAM18908, CNPE-IBUNAM18911, CNPE-IBUNAM18918, CNPE-IBUNAM18919, CNPE-IBUNAM18922, CNPE-IBUNAM18924. |
| H. minckleyi | CNPE-IBUNAM18869, CNPE-IBUNAM18882, CNPE-IBUNAM18917, CNPE-IBUNAM18951, CNPE-IBUNAM18954, CNPE-IBUNAM18959. |
| H. guttatus | CNPE-IBUNAM18871, CNPE-IBUNAM18873, CNPE-IBUNAM18877, CNPE-IBUNAM18880, CNPE-IBUNAM18881, CNPE-IBUNAM18902, CNPE-IBUNAM18904, CNPE-IBUNAM18914, CNPE-IBUNAM18930, CNPE-IBUNAM18935, CNPE-IBUNAM18941, CNPE-IBUNAM18945, CNPE-IBUNAM18950**.** |
| C. xanthicara | CNPE-IBUNAM18862, CNPE-IBUNAM18866, CNPE-IBUNAM18875, CNPE-IBUNAM18885, CNPE-IBUNAM18890, CNPE-IBUNAM18894, CNPE-IBUNAM18910, CNPE-IBUNAM18863, CNPE-IBUNAM18867, CNPE-IBUNAM18907. |
