# Supplementary material for: Trophic analysis of the fish community in the Ciénega Churince, Cuatro Ciénegas, Coahuila
Source: PeerJ. 2017 Sep 4;5:e3637. doi: 10.7717/peerj.3637 (PMC5588786; doi:10.7717/peerj.3637)
Supplement: Supplemental Information 3 [file peerj-05-3637-s003.docx]

Appendix 1

Table 1. Trophic spectrum of *Cyprinodon atrorus*. Percent of number (% N), volumetric (% V), and frequency of occurrence (% FO).

| **Stomach contents** | | | | | | | | | | **%N** | **%V** | **%FO** |
| --- | --- | --- | --- | --- | --- | --- | --- | --- | --- | --- | --- | --- |
| Organic material | | | | | | | | | | 1.94 | 57.62 | 12.16 |
| Food items | | | | | | | | | | 98.06 | 42.38 | 87.84 |
| **Total** | | | | | | | | | | **100.00** | **100.00** | **100.00** |
| Food items | | | | | | | | | | **%N** | **%V** | **%FO** |
| Arthropoda | | | | | | | | | |  |  |  |
|  | Crustacea | | | | | | | | |  |  |  |
|  |  | Maxilopoda | | | | | | | |  |  |  |
|  |  |  | Copepoda 1 | | | | | | | 0.73 | 1.04 | 1.28 |
|  |  |  | Copepoda 2 | | | | | | | 0.34 | 0.48 | 2.37 |
|  |  | Malacostraca | | | | | | | |  |  |  |
|  |  |  | Amphipoda | | | | | | |  |  |  |
|  |  |  |  | Dogielinotidae | | | | | |  |  |  |
|  |  |  |  |  | *Hyalella* | | | | |  |  |  |
|  |  |  |  |  |  | *Hyalella azteca* | | | | 2.71 | 11.05 | 4.55 |
|  |  | Brachipoda | | | | | | | |  |  |  |
|  |  |  | Diplostraca | | | | | | |  |  |  |
|  |  |  |  | Chydoridae | | | | | | 1.51 | 0.97 | 3.64 |
|  |  |  |  |  | Cladocera | | | | | 15.19 | 14.22 | 8.01 |
|  |  | Ostracoda | | | | | | | |  |  |  |
|  |  |  | Podocopida | | | | | | |  |  |  |
|  |  |  |  | Cyprididae | | | | | |  |  |  |
|  |  |  |  |  | *Chlamydotheca* | | | | |  |  |  |
|  |  |  |  |  |  | *Chlamydotheca arcuata* | | | | 0.37 | 0.52 | 2.55 |
|  |  | Chelicerata | | | | | | | |  |  |  |
|  |  |  | Arachnida | | | | | | |  |  |  |
|  |  |  |  | Acari | | | | | |  |  |  |
|  |  |  |  |  | Trombidiformes | | | | |  |  |  |
|  |  |  |  |  |  | Hydrachnidae 1 | | | | 0.16 | 0.22 | 1.09 |
|  |  |  |  |  |  | Hydrachnidae 2 | | | | 0.16 | 0.22 | 1.09 |
|  |  |  |  |  |  | Hydrachnidae 3 | | | | 0.16 | 0.45 | 1.09 |
|  |  |  |  |  |  | Prostigamata 3 | | | | 0.63 | 0.45 | 1.09 |
|  |  |  |  |  | Oribatida 1 | | | | | 0.18 | 0.26 | 1.28 |
|  | Hexapoda | | | | | | | | |  |  |  |
|  |  | Insecta | | | | | | | |  |  |  |
|  |  |  | Ephemenoptera | | | | | | |  |  |  |
|  |  |  |  | Leptohyphidae | | | | | | 0.16 | 0.67 | 1.09 |
|  |  |  | Coleoptera | | | | | | |  |  |  |
|  |  |  |  | Hydrophilidae | | | | | |  |  |  |
|  |  |  |  |  | Berosus | | | | | 0.18 | 3.91 | 1.28 |
|  |  |  | Diptera | | | | | | |  |  |  |
|  |  |  |  | Ceratopogonidae | | | | | |  |  |  |
|  |  |  |  |  | *Bezzia* | | | | |  |  |  |
|  |  |  |  |  |  | *Bezzias sp* | | | | 3.99 | 4.17 | 3.46 |
|  |  |  |  |  | *Culicoides* | | | | | 6.76 | 8.45 | 9.29 |
|  |  |  |  | Chironomidae 1 | | | | | | 43.87 | 27.13 | 13.84 |
|  |  |  |  | Chironomidae 2 | | | | | | 1.36 | 1.94 | 7.10 |
|  |  |  |  | Chironomidae 3 | | | | | | 13.91 | 9.86 | 8.20 |
|  |  |  |  | Chironomidae 5 | | | | | | 0.73 | 1.30 | 1.28 |
|  |  |  |  | Tipulidae | | | | | | 0.50 | 3.01 | 3.46 |
| Mollusca | | | | | | | | | |  |  |  |
|  |  | Gastropoda | | | | | | | |  |  |  |
|  |  |  | Mesogastropoda | | | | | | |  |  |  |
|  |  |  |  | Hydrobiidae | | | | | |  |  |  |
|  |  |  |  |  | Coahuilix | | | | |  |  |  |
|  |  |  |  |  |  | *Coahuilix hubssi* | | | | 0.18 | 0.52 | 1.28 |
| Chordata | | | | | | | | | |  |  |  |
|  | Actinopterygii | | | | | | | | |  |  |  |
|  |  | Teleostei | | |  |  |  |  |  |  |  |  |
|  |  |  | Escama cicloide | | | | | | | 1.93 | 1.86 | 6.92 |
| Plantae | | | | | | | | | |  |  |  |
|  |  |  | Lamiales | | | | | | |  |  |  |
|  |  |  |  | Lentibulariaceae | | | | | |  |  |  |
|  |  |  |  |  | *Urticularia* | | | | |  |  |  |
|  |  |  |  |  |  | *Urticularia obtusa* | | | | 0.50 | 0.48 | 2.37 |
| Plantae 1 | | | | | | | | | | 3.00 | 4.73 | 6.74 |
| Plantae 3 | | | | | | | | | | 0.31 | 0.89 | 2.19 |
| Plantae 9 | | | | | | | | | | 0.16 | 0.22 | 1.09 |
| Plantae 5 | | | | | | | | | | 0.18 | 0.52 | 1.28 |
| N/I 16 | | | | | | | | | | 0.16 | 0.45 | 1.09 |
| **Total** | | | | | | | | | | 100.00 | 100.00 | 100.00 |

Table 2. Trophic spectrum of *Cyprinodon bifasciatus*. Percent of number (% N), volumetric (% V), and frequency of occurrence (% FO).

| **Stomach contents** | | | | | | | | | | **%N** | **%V** | **%FO** |
| --- | --- | --- | --- | --- | --- | --- | --- | --- | --- | --- | --- | --- |
| Organic material | | | | | | | | | | 3.14 | 78.61 | 16.15 |
| Food items | | | | | | | | | | 96.86 | 21.39 | 83.85 |
| **Total** | | | | | | | | | | **100.00** | **100.00** | **100.00** |
| Food items | | | | | | | | | | **%N** | **%V** | **%FO** |
| Arthropoda | | | | | | | | | |  |  |  |
|  | Crustacea | | | | | | | | |  |  |  |
|  |  | Maxilopoda | | | | | | | |  |  |  |
|  |  |  | Copepoda 2 | | | | | | | 39.87 | 6.00 | 6.15 |
|  |  | Malacostraca | | | | | | | |  |  |  |
|  |  |  | Amphipoda | | | | | | |  |  |  |
|  |  |  |  | Dogielinotidae | | | | | |  |  |  |
|  |  |  |  |  | *Hyalella* | | | | |  |  |  |
|  |  |  |  |  |  | *Hyalella azteca* | | | | 0.10 | 0.47 | 0.28 |
|  |  | Brachipoda | | | | | | | |  |  |  |
|  |  |  | Diplostraca | | | | | | |  |  |  |
|  |  |  |  | Chydoridae | | | | | | 6.34 | 8.37 | 12.01 |
|  |  |  | Cladocera | | | | | | | 0.62 | 1.34 | 1.96 |
|  |  | Ostracoda | | | | | | | |  |  |  |
|  |  |  | Podocopida | | | | | | |  |  |  |
|  |  |  |  | Cyprididae | | | | | |  |  |  |
|  |  |  |  |  | *Chlamydotheca* | | | | |  |  |  |
|  |  |  |  |  |  | *Chlamydotheca arcuata* | | | | 18.65 | 13.59 | 14.53 |
|  |  | Chelicerata | | | | | | | |  |  |  |
|  |  |  | Arachnida | | | | | | |  |  |  |
|  |  |  |  | Acari | | | | | |  |  |  |
|  |  |  |  |  | Trombidiformes | | | | |  |  |  |
|  |  |  |  |  |  | Prostigamata 1 | | | | 0.91 | 1.50 | 5.03 |
|  |  |  |  |  |  | Prostigamata 2 | | | | 0.10 | 0.08 | 0.28 |
|  |  |  |  | Aranae | | | | | | 0.19 | 1.26 | 1.12 |
|  | Hexapoda | | | | | | | | |  |  |  |
|  |  | Insecta | | | | | | | |  |  |  |
|  |  |  | Diptera | | | | | | |  |  |  |
|  |  |  |  | Ceratopogonidae | | | | | |  |  |  |
|  |  |  |  |  | *Culicoides* | | | | | 3.10 | 7.50 | 8.10 |
|  |  |  |  | Chironomidae 1 | | | | | | 14.40 | 19.43 | 11.45 |
|  |  |  |  | Chironomidae 2 | | | | | | 0.19 | 0.32 | 1.12 |
|  |  |  |  | Chironomidae 3 | | | | | | 0.14 | 0.24 | 0.84 |
|  |  |  |  | Tipulidae 1 | | | | | | 0.76 | 3.00 | 3.07 |
|  |  |  |  | Tipulidae 4 | | | | | | 0.38 | 4.11 | 1.12 |
|  |  | Insecta 21 | | | | | | | | 0.05 | 2.76 | 0.28 |
| Mollusca | | | | | | | | | |  |  |  |
|  |  | Gastropoda | | | | | | | |  |  |  |
|  |  |  | Mesogastropoda | | | | | | |  |  |  |
|  |  |  |  | Hydrobiidae | | | | | |  |  |  |
|  |  |  |  |  | Coahuilix | | | | |  |  |  |
|  |  |  |  |  |  | Coahuilix hubssi | | | | 0.19 | 1.26 | 1.12 |
|  |  |  |  |  | *Pyrgulopsis* | | | | |  |  |  |
|  |  |  |  |  |  | *Pyrgulopsis manantiali* | | | | 0.57 | 1.90 | 0.84 |
| Chordata | | | | | | | | | |  |  |  |
|  | Actinopterygii | | | | | | | | |  |  |  |
|  |  | Teleostei | | |  |  |  |  |  |  |  |  |
|  |  |  | Escama cicloide | | | | | | | 2.77 | 2.84 | 8.38 |
|  |  |  | Escama ctenoide | | | | | | | 0.10 | 0.16 | 0.56 |
|  |  |  | Vertebras | | | | | | | 0.19 | 0.32 | 1.12 |
| Nematoda | | | | | | | | | |  |  |  |
|  |  | Adenoforea | | | | | | | |  |  |  |
|  |  |  | Monhysterida | | | | | | |  |  |  |
|  |  |  |  | Siphonolaimidae | | | | | | 0.29 | 0.79 | 1.68 |
| Plantae | | | | | | | | | |  |  |  |
|  |  |  | Lamiales | | | | | | |  |  |  |
|  |  |  |  | Lentibulariaceae | | | | | |  |  |  |
|  |  |  |  |  | *Urticularia* | | | | |  |  |  |
|  |  |  |  |  |  | *Urticularia obtusa* | | | | 4.77 | 8.53 | 8.10 |
| Plantae 2 | | | | | | | | | | 2.58 | 8.06 | 6.98 |
| Plantae 3 | | | | | | | | | | 2.29 | 5.06 | 1.96 |
| Plantae 4 | | | | | | | | | | 0.19 | 0.32 | 0.56 |
| Plantae 6 | | | | | | | | | | 0.10 | 0.16 | 0.56 |
| Plantae 7 | | | | | | | | | | 0.19 | 0.63 | 0.84 |
| **Total** | | | | | | | | | | **100.00** | **100.00** | **100.00** |

Table 3. Trophic spectrum of *Gambusia marshi* of Laguna intermedia. Percent of number (% N), volumetric (% V), and frequency of occurrence (% FO).

| **Stomach contents** | | | | | | | | | | **%N** | **%V** | **%FO** |
| --- | --- | --- | --- | --- | --- | --- | --- | --- | --- | --- | --- | --- |
| Organic material | | | | | | | | | | 10.93 | 57.33 | 24.12 |
| Food items | | | | | | | | | | 89.07 | 42.67 | 75.88 |
| **Total** | | | | | | | | | | **100.00** | **100.00** | **100.00** |
| Food items | | | | | | | | | | **%N** | **%V** | **%FO** |
| Arthropoda | | | | | | | | | |  |  |  |
|  | Crustacea | | | | | | | | |  |  |  |
|  |  | Maxilopoda | | | | | | | |  |  |  |
|  |  |  | Copepoda 1 | | | | | | | 0.40 | 0.14 | 1.03 |
|  |  |  | Copepoda 2 | | | | | | | 3.56 | 1.24 | 5.13 |
|  |  | Malacostraca | | | | | | | |  |  |  |
|  |  |  | Amphipoda | | | | | | |  |  |  |
|  |  |  |  | Dogielinotidae | | | | | |  |  |  |
|  |  |  |  |  | *Hyalella* | | | | |  |  |  |
|  |  |  |  |  |  | *Hyalella azteca* | | | | 17.82 | 36.15 | 10.77 |
|  |  | Ostracoda | | | | | | | |  |  |  |
|  |  |  | Podocopida | | | | | | |  |  |  |
|  |  |  |  | Cyprididae | | | | | |  |  |  |
|  |  |  |  |  | *Chlamydotheca* | | | | |  |  |  |
|  |  |  |  |  |  | *Chlamydotheca arcuata* | | | | 2.97 | 0.34 | 1.54 |
|  |  | Chelicerata | | | | | | | |  |  |  |
|  |  |  | Arachnida | | | | | | |  |  |  |
|  |  |  |  | Acari | | | | | |  |  |  |
|  |  |  |  |  | Trombidiformes | | | | |  |  |  |
|  |  |  |  |  |  | Hidrachnidae 1 | | | | 2.18 | 0.76 | 3.59 |
|  |  |  |  |  |  | Prostigamata 1 | | | | 1.39 | 1.17 | 3.59 |
|  |  |  |  |  |  | Prostigamata 2 | | | | 0.20 | 0.07 | 0.51 |
|  |  |  |  |  |  | Prostigamata 3 | | | | 0.20 | 0.14 | 0.51 |
|  |  |  |  | Aranae 1 | | | | | | 1.58 | 2.20 | 4.10 |
|  | Hexapoda | | | | | | | | |  |  |  |
|  |  | Insecta | | | | | | | |  |  |  |
|  |  |  | Ephemenoptera 1 | | | | | | | 0.20 | 0.69 | 0.51 |
|  |  |  |  | Leptohyphidae 1 | | | | | | 0.99 | 2.41 | 4.62 |
|  |  |  | Odonata 2 | | | | | | | 1.78 | 2.06 | 1.54 |
|  |  |  | Coleoptera | | | | | | |  |  |  |
|  |  |  |  | Hydrophilidae | | | | | |  |  |  |
|  |  |  |  |  | Berosus | | | | | 0.79 | 4.12 | 2.05 |
|  |  |  | Diptera | | | | | | |  |  |  |
|  |  |  |  | Simulidae 1 | | | | | | 2.38 | 0.82 | 1.54 |
|  |  |  |  | Ceratopogonidae | | | | | |  |  |  |
|  |  |  |  |  | *Bezzia* | | | | |  |  |  |
|  |  |  |  |  |  | *Bezzias sp* | | | | 0.20 | 0.34 | 0.51 |
|  |  |  |  |  | *Culicoides* | | | | | 20.79 | 13.75 | 12.82 |
|  |  |  |  | Chironomidae 1 | | | | | | 6.53 | 4.19 | 8.72 |
|  |  |  |  | Chironomidae 2 | | | | | | 0.40 | 0.14 | 1.03 |
|  |  |  |  | Tipulidae 1 | | | | | | 1.39 | 4.95 | 3.59 |
|  |  |  | Hemiptera | | | | | | |  |  |  |
|  |  |  |  | Hebridae | | | | | |  |  |  |
|  |  |  |  |  | *Hebrus* | | | | | 1.58 | 4.95 | 2.05 |
|  |  |  | Hymenoptera 1 | | | | | | | 0.79 | 5.50 | 2.05 |
|  |  |  |  | Formacidae 1 | | | | | | 2.38 | 4.26 | 6.15 |
|  |  |  |  | Formacidae 2 | | | | | | 0.79 | 1.92 | 2.05 |
|  |  | Insecta 9 | | | | | | | | 0.79 | 0.34 | 2.05 |
|  |  | Insecta 20 | | | | | | | | 0.20 | 1.03 | 0.51 |
|  |  | Insecta 25 | | | | | | | | 0.40 | 0.69 | 1.03 |
| Mollusca | | | | | | | | | |  |  |  |
|  |  | Gastropoda | | | | | | | |  |  |  |
|  |  |  | Mesogastropoda | | | | | | |  |  |  |
|  |  |  |  | Hydrobiidae | | | | | |  |  |  |
|  |  |  |  |  | *Pyrgulopsis* | | | | |  |  |  |
|  |  |  |  |  |  | *Pyrgulopsis manantiali* | | | | 0.20 | 0.07 | 0.51 |
| Chordata | | | | | | | | | |  |  |  |
|  | Actinopterygii | | | | | | | | |  |  |  |
|  |  | Teleostei | | |  |  |  |  |  |  |  |  |
|  |  |  | Cyprinodontiformes | | | | | | |  |  |  |
|  |  |  |  | Poeciliidae | | | | | |  |  |  |
|  |  |  |  |  | *Gambusia* | | | | |  |  |  |
|  |  |  |  |  |  | *Gambusia marshi* | | | | 0.59 | 3.09 | 1.54 |
|  |  |  | Escama cicloide | | | | | | | 4.55 | 1.51 | 8.21 |
| Nematoda | | | | | | | | | |  |  |  |
|  |  | Adenoforea | | | | | | | |  |  |  |
|  |  |  | Monhysterida | | | | | | |  |  |  |
|  |  |  |  | Siphonolaimidae | | | | | | 1.19 | 0.41 | 3.08 |
| Plantae | | | | | | | | | |  |  |  |
| Plantae 4 | | | | | | | | | | 0.20 | 0.14 | 0.51 |
| Plantae 9 | | | | | | | | | | 19.80 | 0.14 | 0.51 |
| N/I 9 | | | | | | | | | | 0.79 | 0.27 | 2.05 |
| **Total** | | | | | | | | | | **100.00** | **100.00** | **100.00** |

Table 4. Trophic spectrum of *Gambusia marshi* of Poza Churince. Percent of number (% N), volumetric (% V), and frequency of occurrence (% FO).

| **Stomach contents** | | | | | | | | | | **%N** | **%V** | **%FO** |
| --- | --- | --- | --- | --- | --- | --- | --- | --- | --- | --- | --- | --- |
| Organic material | | | | | | | | | | 1.72 | 64.78 | 14.37 |
| Food items | | | | | | | | | | 98.28 | 35.22 | 85.63 |
| **Total** | | | | | | | | | | **100.00** | **100.00** | **100.00** |
| Food items | | | | | | | | | | **%N** | **%V** | **%FO** |
| Arthropoda | | | | | | | | | |  |  |  |
|  | Crustacea | | | | | | | | |  |  |  |
|  |  | Maxilopoda | | | | | | | |  |  |  |
|  |  |  | Copepoda 1 | | | | | | | 0.05 | 0.10 | 0.43 |
|  |  |  | Copepoda 2 | | | | | | | 0.11 | 0.15 | 0.65 |
|  |  | Malacostraca | | | | | | | |  |  |  |
|  |  |  | Amphipoda | | | | | | |  |  |  |
|  |  |  |  | Dogielinotidae | | | | | |  |  |  |
|  |  |  |  |  | *Hyalella* | | | | |  |  |  |
|  |  |  |  |  |  | *Hyalella azteca* | | | | 0.34 | 1.68 | 2.81 |
|  |  | Brachipoda | | | | | | | |  |  |  |
|  |  |  | Diplostraca | | | | | | |  |  |  |
|  |  |  |  | Chydoridae | | | | | | 4.10 | 3.91 | 10.15 |
|  |  |  | Cladocera | | | | | | | 0.18 | 0.66 | 1.73 |
|  |  | Ostracoda | | | | | | | |  |  |  |
|  |  |  | Podocopida | | | | | | |  |  |  |
|  |  |  |  | Cyprididae | | | | | |  |  |  |
|  |  |  |  |  | *Chlamydotheca* | | | | |  |  |  |
|  |  |  |  |  |  | *Chlamydotheca arcuata* | | | | 13.38 | 5.38 | 9.94 |
|  |  | Chelicerata | | | | | | | |  |  |  |
|  |  |  | Arachnida | | | | | | |  |  |  |
|  |  |  |  | Acari | | | | | |  |  |  |
|  |  |  |  |  | Trombidiformes | | | | |  |  |  |
|  |  |  |  |  |  | Hidrachnidae 1 | | | | 0.02 | 0.05 | 0.22 |
|  |  |  |  |  |  | Hidrachnidae 2 | | | | 0.07 | 0.15 | 0.65 |
|  |  |  |  |  |  | Prostigamata 1 | | | | 1.18 | 2.44 | 3.89 |
|  |  |  |  |  |  | Prostigamata 2 | | | | 0.18 | 0.20 | 0.86 |
|  |  |  |  |  |  | Prostigamata 3 | | | | 0.11 | 0.25 | 0.65 |
|  |  |  |  | Aranae 1 | | | | | | 1.50 | 2.59 | 1.94 |
|  |  |  |  | Aranae 2 | | | | | | 0.36 | 0.20 | 0.43 |
|  | Hexapoda | | | | | | | | |  |  |  |
|  |  | Insecta | | | | | | | |  |  |  |
|  |  |  | Ephemenoptera 1 | | | | | | |  |  |  |
|  |  |  |  | Leptohyphidae 1 | | | | | | 0.20 | 1.37 | 1.94 |
|  |  |  | Coleoptera | | | | | | |  |  |  |
|  |  |  |  | Hydrophilidae | | | | | |  |  |  |
|  |  |  |  |  | Berosus | | | | | 0.09 | 0.20 | 0.86 |
|  |  |  | Diptera | | | | | | |  |  |  |
|  |  |  |  | Ceratopogonidae | | | | | |  |  |  |
|  |  |  |  |  | *Culicoides* | | | | | 10.61 | 17.67 | 11.88 |
|  |  |  |  | Chironomidae 1 | | | | | | 1.41 | 3.35 | 6.48 |
|  |  |  |  | Chironomidae 2 | | | | | | 0.25 | 0.10 | 0.22 |
|  |  |  |  | Chironomidae 3 | | | | | | 0.09 | 0.20 | 0.86 |
|  |  |  |  | Chironomidae 5 | | | | | | 0.18 | 2.34 | 0.43 |
|  |  |  |  | Tipulidae 1 | | | | | | 1.75 | 5.49 | 4.75 |
|  |  |  |  | Tipulidae 2 | | | | | | 0.05 | 1.02 | 0.43 |
|  |  |  |  | Tipulidae 5 | | | | | | 0.02 | 0.51 | 0.22 |
|  |  |  | Hymenoptera | | | | | | |  |  |  |
|  |  |  |  | Braconidae | | | | | | 0.20 | 6.86 | 1.94 |
|  |  |  |  | Formacidae 1 | | | | | | 0.20 | 7.31 | 1.08 |
|  |  |  |  | Formacidae 2 | | | | | | 0.20 | 2.49 | 1.94 |
|  |  |  | Heteroptera | | | | | | |  |  |  |
|  |  |  |  | Mesoveliidae | | | | | | 0.05 | 1.83 | 0.43 |
|  |  | Insecta 5 | | | | | | | | 0.23 | 0.86 | 0.86 |
|  |  | Insecta 7 | | | | | | | | 0.02 | 0.10 | 0.22 |
|  |  | Insecta 18 | | | | | | | | 0.05 | 0.20 | 0.43 |
|  |  | Insecta 22 | | | | | | | | 0.05 | 4.06 | 0.43 |
| Chordata | | | | | | | | | |  |  |  |
|  | Actinopterygii | | | | | | | | |  |  |  |
|  |  | Teleostei | | |  |  |  |  |  |  |  |  |
|  |  |  | Radios | | | | | | | 0.05 | 0.10 | 0.43 |
|  |  |  | Escama ctenoide | | | | | | | 0.79 | 1.27 | 5.40 |
|  |  |  | Huevos | | | | | | | 0.05 | 2.03 | 0.43 |
| Plantae | | | | | | | | | |  |  |  |
|  |  |  | Lamiales | | | | | | |  |  |  |
|  |  |  |  | Lentibulariaceae | | | | | |  |  |  |
|  |  |  |  |  | *Urticularia* | | | | |  |  |  |
|  |  |  |  |  |  | *Urticularia obtusa* | | | | 0.66 | 1.12 | 2.16 |
| Plantae 1 | | | | | | | | | | 0.43 | 0.86 | 1.51 |
| Plantae 4 | | | | | | | | | | 4.13 | 9.40 | 7.34 |
| Plantae 2 | | | | | | | | | | 0.63 | 2.18 | 2.59 |
| Plantae 5 | | | | | | | | | | 0.11 | 0.41 | 1.08 |
| Plantae 6 | | | | | | | | | | 0.36 | 0.61 | 2.59 |
| Plantae 7 | | | | | | | | | | 0.45 | 1.02 | 0.22 |
| Plantae 8 | | | | | | | | | | 0.02 | 0.15 | 0.22 |
| Plantae 9 | | | | | | | | | | 55.03 | 6.91 | 5.83 |
| Plantae 10 | | | | | | | | | | 0.05 | 0.20 | 0.43 |
| **Total** | | | | | | | | | | **100.00** | **100.00** | **100.00** |

Table 5. Trophic spectrum of *Lepomis macrochirus*. Percent of number (% N), volumetric (% V), and frequency of occurrence (% FO).

| **Stomach contents** | | | | | | | | | | **%N** | **%V** | **%FO** |
| --- | --- | --- | --- | --- | --- | --- | --- | --- | --- | --- | --- | --- |
| Organic material | | | | | | | | | | 1.23 | 24.67 | 13.68 |
| Food items | | | | | | | | | | 98.77 | 75.33 | 86.32 |
| **Total** | | | | | | | | | | **100.00** | **100.00** | **100.00** |
| Food items | | | | | | | | | | **%N** | **%V** | **%FO** |
| Arthropoda | | | | | | | | | |  |  |  |
|  | Crustacea | | | | | | | | |  |  |  |
|  |  | Maxilopoda | | | | | | | |  |  |  |
|  |  |  | Copepoda 1 | | | | | | | 0.38 | 0.59 | 2.44 |
|  |  |  | Copepoda 2 | | | | | | | 0.19 | 0.29 | 2.44 |
|  |  | Malacostraca | | | | | | | |  |  |  |
|  |  |  | Amphipoda | | | | | | |  |  |  |
|  |  |  |  | Dogielinotidae | | | | | |  |  |  |
|  |  |  |  |  | *Hyalella* | | | | |  |  |  |
|  |  |  |  |  |  | *Hyalella azteca* | | | | 13.74 | 24.93 | 14.63 |
|  |  | Brachipoda | | | | | | | |  |  |  |
|  |  |  | Diplostraca | | | | | | |  |  |  |
|  |  |  |  | Chydoridae | | | | | | 0.38 | 0.29 | 2.44 |
|  |  | Ostracoda | | | | | | | |  |  |  |
|  |  |  | Podocopida | | | | | | |  |  |  |
|  |  |  |  | Cyprididae | | | | | |  |  |  |
|  |  |  |  |  | *Chlamydotheca* | | | | |  |  |  |
|  |  |  |  |  |  | *Chlamydotheca arcuata* | | | | 3.24 | 4.42 | 6.10 |
|  |  | Chelicerata | | | | | | | |  |  |  |
|  |  |  | Arachnida | | | | | | |  |  |  |
|  |  |  |  | Acari | | | | | |  |  |  |
|  |  |  |  |  | Trombidiformes | | | | |  |  |  |
|  |  |  |  |  |  | Hydrachnidae 1 | | | | 0.29 | 0.44 | 3.66 |
|  |  |  |  |  |  | Prostigamata 1 | | | | 0.57 | 0.29 | 2.44 |
|  | Hexapoda | | | | | | | | |  |  |  |
|  |  | Insecta | | | | | | | |  |  |  |
|  |  |  | Ephemenoptera | | | | | | |  |  |  |
|  |  |  |  | Leptohyphidae 1 | | | | | | 1.24 | 7.08 | 6.10 |
|  |  |  | Diptera | | | | | | |  |  |  |
|  |  |  |  | Ceratopogonidae | | | | | |  |  |  |
|  |  |  |  |  | *Culicoides* | | | | | 1.81 | 14.45 | 2.44 |
|  |  |  |  | Chironomidae 1 | | | | | | 35.88 | 14.31 | 13.41 |
|  |  |  |  | Chironomidae 2 | | | | | | 15.27 | 4.28 | 6.10 |
|  |  |  |  | Chironomidae 3 | | | | | | 22.04 | 10.03 | 14.63 |
|  |  |  |  | Tipulidae | | | | | | 2.48 | 4.13 | 6.10 |
|  |  |  | Hemiptera | | | | | | |  |  |  |
|  |  |  |  | Nepidae | | | | | |  |  |  |
|  |  |  |  |  | *Ranatra* | | | | | 0.19 | 1.47 | 2.44 |
|  |  |  |  |  |  |  |  |  |  |  |  |  |
| Chordata | | | | | | | | | |  |  |  |
|  | Actinopterygii | | | | | | | | |  |  |  |
|  |  | Teleostei | | |  |  |  |  |  |  |  |  |
|  |  |  | Vertebras | | | | | | | 0.38 | 1.47 | 1.22 |
|  |  |  | Escama cicloide | | | | | | | 1.24 | 2.21 | 7.32 |
| Nematoda | | | | | | | | | |  |  |  |
|  |  | Adenoforea | | | | | | | |  |  |  |
|  |  |  | Monhysterida | | | | | | |  |  |  |
|  |  |  |  | Siphonolaimidae | | | | | | 0.38 | 8.85 | 2.44 |
| Plantae 5 | | | | | | | | | | 0.29 | 0.44 | 3.66 |
| **Total** | | | | | | | | | | **100.00** | **100.00** | **100.00** |

Table 6. Trophic spectrum of *Lepomis megalotis*. Percent of number (% N), volumetric (% V), and frequency of occurrence (% FO).

| **Stomach contents** | | | | | | | | | | **%N** | **%V** | **%FO** |
| --- | --- | --- | --- | --- | --- | --- | --- | --- | --- | --- | --- | --- |
| Organic material | | | | | | | | | | 1.52 | 40.36 | 14.75 |
| Food items | | | | | | | | | | 98.48 | 59.64 | 85.25 |
| **Total** | | | | | | | | | | **100.00** | **100.00** | **100.00** |
| Food items | | | | | | | | | | **%N** | **%V** | **%FO** |
| Arthropoda | | | | | | | | | |  |  |  |
|  | Crustacea | | | | | | | | |  |  |  |
|  |  | Malacostraca | | | | | | | |  |  |  |
|  |  |  | Amphipoda | | | | | | |  |  |  |
|  |  |  |  | Dogielinotidae | | | | | |  |  |  |
|  |  |  |  |  | *Hyalella* | | | | |  |  |  |
|  |  |  |  |  |  | *Hyalella azteca* | | | | 48.21 | 30.54 | 19.23 |
|  |  | Brachipoda | | | | | | | |  |  |  |
|  |  |  | Cladocera | | | | | | | 4.44 | 7.78 | 1.92 |
|  |  | Ostracoda | | | | | | | |  |  |  |
|  |  |  | Podocopida | | | | | | |  |  |  |
|  |  |  |  | Cyprididae | | | | | |  |  |  |
|  |  |  |  |  | *Chlamydotheca* | | | | |  |  |  |
|  |  |  |  |  |  | *Chlamydotheca arcuata* | | | | 0.68 | 1.50 | 3.85 |
|  |  | Chelicerata | | | | | | | |  |  |  |
|  |  |  | Arachnida | | | | | | |  |  |  |
|  |  |  |  | Aranae 1 | | | | | | 0.17 | 1.50 | 1.92 |
|  | Hexapoda | | | | | | | | |  |  |  |
|  |  | Insecta | | | | | | | |  |  |  |
|  |  |  | Ephemeroptera | | | | | | |  |  |  |
|  |  |  |  | Caenidae | | | | | |  |  |  |
|  |  |  |  |  | Caenis | | | | | 24.62 | 32.34 | 11.54 |
|  |  |  |  | Leptohyphidae 1 | | | | | | 0.17 | 0.30 | 1.92 |
|  |  |  | Coleoptera | | | | | | |  |  |  |
|  |  |  |  | Hydrophilidae | | | | | |  |  |  |
|  |  |  |  |  | Berosus | | | | | 0.17 | 0.90 | 1.92 |
|  |  |  | Odonata 2 | | | | | | | 7.35 | 4.49 | 7.69 |
|  |  |  | Diptera | | | | | | |  |  |  |
|  |  |  |  | Ceratopogonidae | | | | | |  |  |  |
|  |  |  |  |  | *Culicoides* | | | | | 4.27 | 3.59 | 5.77 |
|  |  |  |  | Chironomidae 1 | | | | | | 1.88 | 3.29 | 9.62 |
|  |  |  |  | Chironomidae 5 | | | | | | 0.17 | 0.30 | 1.92 |
|  |  |  | Hymenoptera | | | | | | |  |  |  |
|  |  |  |  | Formacidae 1 | | | | | | 0.17 | 0.60 | 1.92 |
|  |  |  |  | Formacidae 2 | | | | | | 0.51 | 2.99 | 1.92 |
|  |  |  | Heteroptera | | | | | | |  |  |  |
|  |  |  |  | Mesoveliidae | | | | | | 0.17 | 0.30 | 1.92 |
| Mollusca | | | | | | | | | |  |  |  |
|  |  | Gastropoda | | | | | | | |  |  |  |
|  |  |  | Mesogastropoda | | | | | | |  |  |  |
|  |  |  |  | Hydrobiidae | | | | | |  |  |  |
|  |  |  |  |  | *Pyrgulopsis* | | | | |  |  |  |
|  |  |  |  |  |  | *Pyrgulopsis manantiali* | | | | 0.68 | 2.40 | 5.77 |
|  |  | Branquea | | | | *Pyrgulopsis manantiali* | | | | 0.17 | 0.30 | 1.92 |
|  |  |  |  |  | Coahuilix | | | | |  |  |  |
|  |  |  |  |  |  | Coahuilix hubssi | | | | 0.17 | 0.60 | 1.92 |
| Chordata | | | | | | | | | |  |  |  |
|  | Actinopterygii | | | | | | | | |  |  |  |
|  |  | Teleostei | | |  |  |  |  |  |  |  |  |
|  |  |  | Dientes | | | | | | | 0.51 | 0.60 | 3.85 |
|  |  |  | Vertebras | | | | | | | 3.08 | 1.80 | 1.92 |
|  |  |  | Escama cicloide | | | | | | | 0.17 | 0.30 | 1.92 |
| Nematoda | | | | | | | | | |  |  |  |
|  |  | Adenoforea | | | | | | | |  |  |  |
|  |  |  | Monhysterida | | | | | | |  |  |  |
|  |  |  |  | Siphonolaimidae | | | | | | 1.37 | 0.60 | 3.85 |
| Plantae 5 | | | | | | | | | | 0.51 | 1.50 | 1.92 |
| Plantae 7 | | | | | | | | | | 0.17 | 0.60 | 1.92 |
| Plantae 3 | | | | | | | | | | 0.17 | 0.90 | 1.92 |
| **Total** | | | | | | | | | | **100.00** | **100.00** | **100.00** |

Table 7. Trophic spectrum of *Micropterus salmoides*. Percent of number (% N), volumetric (% V), and frequency of occurrence (% FO).

| **Stomach contents** | | | | | | | | | | **%N** | **%V** | **%FO** |
| --- | --- | --- | --- | --- | --- | --- | --- | --- | --- | --- | --- | --- |
| Organic material | | | | | | | | | | 4.25 | 28.75 | 16.92 |
| Food items | | | | | | | | | | 95.75 | 71.25 | 83.08 |
| **Total** | | | | | | | | | | **100.00** | **100.00** | **100.00** |
| Food items | | | | | | | | | | **%N** | **%V** | **%FO** |
| Arthropoda | | | | | | | | | |  |  |  |
|  | Crustacea | | | | | | | | |  |  |  |
|  | Crustacea | | | | | | | | |  |  |  |
|  |  | Maxilopoda | | | | | | | |  |  |  |
|  |  |  | Copepoda 1 | | | | | | | 2.79 | 1.19 | 3.70 |
|  |  | Malacostraca | | | | | | | |  |  |  |
|  |  |  | Amphipoda | | | | | | |  |  |  |
|  |  |  |  | Dogielinotidae | | | | | |  |  |  |
|  |  |  |  |  | *Hyalella* | | | | |  |  |  |
|  |  |  |  |  |  | *Hyalella azteca* | | | | 8.47 | 27.76 | 12.04 |
|  |  | Brachipoda | | | | | | | |  |  |  |
|  |  |  | Diplostraca | | | | | | |  |  |  |
|  |  |  |  | Chydoridae | | | | | | 1.45 | 0.36 | 2.78 |
|  |  |  | Cladocera | | | | | | | 13.64 | 2.27 | 1.85 |
|  |  | Ostracoda | | | | | | | |  |  |  |
|  |  |  | Podocopida | | | | | | |  |  |  |
|  |  |  |  | Cyprididae | | | | | |  |  |  |
|  |  |  |  |  | *Chlamydotheca* | | | | |  |  |  |
|  |  |  |  |  |  | *Chlamydotheca arcuata* | | | | 0.93 | 0.36 | 3.24 |
|  |  | Chelicerata | | | | | | | |  |  |  |
|  |  |  | Arachnida | | | | | | |  |  |  |
|  |  |  |  | Acari | | | | | |  |  |  |
|  |  |  |  |  | Trombidiformes | | | | |  |  |  |
|  |  |  |  |  |  | Hygrobatidae | | | | 9.04 | 0.30 | 1.01 |
|  |  |  |  |  | Acariformes 1 | | | | | 1.80 | 0.11 | 1.30 |
|  | Hexapoda | | | | | | | | |  |  |  |
|  |  | Insecta | | | | | | | |  |  |  |
|  |  |  | Ephemeroptera 2 | | | | | | | 1.17 | 3.00 | 3.00 |
|  |  |  |  | Leptohyphidae 1 | | | | | | 1.50 | 10.00 | 6.94 |
|  |  |  | Coleoptera 1 | | | | | | | 0.21 | 2.00 | 0.50 |
|  |  |  |  | Belidae 2 | | | | | | 0.10 | 0.52 | 0.46 |
|  |  |  | Odonata 1 | | | | | | | 0.50 | 5.00 | 1.00 |
|  |  |  |  | Aeshnidae | | | | | | 1.00 | 0.67 | 1.63 |
|  |  |  |  | Anisoptera | | | | | | 0.98 | 2.69 | 0.39 |
|  |  |  |  | Libellulidae 2 | | | | | | 0.10 | 0.32 | 0.50 |
|  |  |  |  | Libellulidae 3 | | | | | | 0.43 | 1.19 | 0.39 |
|  |  |  | Diptera | | | | | | |  |  |  |
|  |  |  |  | Ceratopogonidae | | | | | |  |  |  |
|  |  |  |  |  | *Bezzia* | | | | |  |  |  |
|  |  |  |  |  |  | *Bezzias sp* | | | | 0.62 | 0.31 | 1.85 |
|  |  |  |  |  | *Culicoides* | | | | | 23.24 | 8.00 | 7.87 |
|  |  |  |  | Chironomidae 1 | | | | | | 9.74 | 4.30 | 9.20 |
|  |  |  |  | Chironomidae 2 | | | | | | 5.48 | 1.65 | 4.63 |
|  |  |  |  | Chironomidae 3 | | | | | | 0.31 | 0.26 | 0.46 |
|  |  |  |  | Chironomidae 4 | | | | | | 0.80 | 0.50 | 1.45 |
|  |  |  |  | Chironomidae 5 | | | | | | 1.03 | 0.36 | 3.24 |
|  |  |  |  | Tipulidae 1 | | | | | | 6.92 | 3.97 | 6.48 |
|  |  |  |  | Tipulidae 3 | | | | | | 0.21 | 1.55 | 0.93 |
|  |  |  | Hymenoptera | | | | | | |  |  |  |
|  |  |  |  | Formacidae 1 | | | | | | 1.65 | 0.88 | 1.85 |
|  |  |  |  | Formacidae 2 | | | | | | 0.31 | 0.77 | 1.39 |
|  |  |  | Lepidoptera 1 | | | | | | | 0.21 | 4.13 | 0.93 |
|  |  | Insecta 3 | | | | | | | | 0.10 | 0.52 | 0.46 |
|  |  | Insecta 6 | | | | | | | | 0.21 | 0.31 | 0.93 |
|  |  | Insecta 7 | | | | | | | | 0.21 | 2.06 | 0.93 |
|  |  | Insecta 19 | | | | | | | | 0.52 | 0.98 | 2.31 |
|  |  | Insecta 23 | | | | | | | | 0.31 | 0.31 | 1.39 |
|  |  | Larva de Insecto | | | | | | | | 0.21 | 0.21 | 0.93 |
| Chordata | | | | | | | | | |  |  |  |
|  | Actinopterygii | | | | | | | | |  |  |  |
|  |  | Teleostei | | |  |  |  |  |  |  |  |  |
|  |  |  | Cyprinodontiformes | | | | | | |  |  |  |
|  |  |  |  | Poeciliidae | | | | | |  |  |  |
|  |  |  |  |  | *Gambusia* | | | | |  |  |  |
|  |  |  |  |  |  | *Gambusia marshi* | | | | 0.21 | 6.19 | 0.46 |
|  |  |  | Vertebras | | | | | | | 0.21 | 1.55 | 0.93 |
|  |  |  | Escama cicloide | | | | | | | 0.72 | 0.05 | 1.85 |
|  |  |  | Escama ctenoide | | | | | | | 1.03 | 0.41 | 2.78 |
| Plantae 2 | | | | | | | | | | 0.41 | 1.24 | 0.93 |
| Plantae 5 | | | | | | | | | | 0.52 | 0.26 | 2.31 |
| N/I 14 | | | | | | | | | | 0.72 | 1.50 | 2.78 |
| **Total** | | | | | | | | | | **100.00** | **100.00** | **100.00** |

Table 8. Trophic spectrum of *Herichthys minckleyi*. Percent of number (% N), volumetric (% V), and frequency of occurrence (% FO).

| **Stomach contents** | | | | | | | | | | **%N** | **%V** | **%FO** |
| --- | --- | --- | --- | --- | --- | --- | --- | --- | --- | --- | --- | --- |
| Organic material | | | | | | | | | | 0.41 | 12.95 | 7.46 |
| Food items | | | | | | | | | | 99.59 | 87.05 | 92.54 |
| **Total** | | | | | | | | | | **100.00** | **100.00** | **100.00** |
| Food items | | | | | | | | | | **%N** | **%V** | **%FO** |
| Arthropoda | | | | | | | | | |  |  |  |
|  | Crustacea | | | | | | | | |  |  |  |
|  |  | Brachipoda | | | | | | | |  |  |  |
|  |  |  | Diplostraca | | | | | | |  |  |  |
|  |  |  |  | Chydoridae | | | | | | 0.33 | 0.52 | 3.23 |
|  |  | Ostracoda | | | | | | | |  |  |  |
|  |  |  | Podocopida | | | | | | |  |  |  |
|  |  |  |  | Cyprididae | | | | | |  |  |  |
|  |  |  |  |  | *Chlamydotheca* | | | | |  |  |  |
|  |  |  |  |  |  | *Chlamydotheca arcuata* | | | | 1.39 | 3.39 | 8.06 |
|  |  | Chelicerata | | | | | | | |  |  |  |
|  |  |  | Arachnida | | | | | | |  |  |  |
|  |  |  |  | Acari | | | | | |  |  |  |
|  |  |  |  |  | Trombidiformes | | | | |  |  |  |
|  |  |  |  |  |  | Prostigamata 1 | | | | 0.82 | 1.57 | 3.23 |
|  |  |  |  | Aranae 1 | | | | | | 0.16 | 0.52 | 1.61 |
|  | Hexapoda | | | | | | | | |  |  |  |
|  |  | Insecta | | | | | | | |  |  |  |
|  |  |  | Ephemeroptera | | | | | | |  |  |  |
|  |  |  |  | Leptohyphidae 1 | | | | | | 0.25 | 7.83 | 4.84 |
|  |  |  | Diptera | | | | | | |  |  |  |
|  |  |  |  | Ceratopogonidae | | | | | |  |  |  |
|  |  |  |  | Chironomidae 1 | | | | | | 0.57 | 1.83 | 4.84 |
|  |  |  |  | Chironomidae 2 | | | | | | 0.08 | 0.26 | 1.61 |
|  |  |  |  | Chironomidae 3 | | | | | | 0.33 | 1.04 | 1.61 |
|  |  |  |  | Tipulidae 1 | | | | | | 0.16 | 0.78 | 3.23 |
|  |  |  | Hymenoptera | | | | | | |  |  |  |
|  |  |  |  | Formacidae 1 | | | | | | 0.08 | 0.52 | 1.61 |
|  |  |  |  | Formacidae 2 | | | | | | 0.33 | 1.04 | 3.23 |
| Mollusca | | | | | | | | | |  |  |  |
|  |  | Gastropoda | | | | | | | |  |  |  |
|  |  |  | Mesogastropoda | | | | | | |  |  |  |
|  |  |  |  | Hydrobiidae 2 | | | | | | 1.56 | 2.35 | 1.61 |
|  |  |  |  | Hydrobiidae 4 | | | | | | 2.13 | 6.79 | 4.84 |
|  | Branquea | | | Hydrobiidae 3 | | | | | | 2.79 | 0.78 | 1.61 |
|  |  |  |  |  | *Pyrgulopsis* | | | | |  |  |  |
|  |  |  |  |  |  | *Pyrgulopsis manantiali* | | | | 44.18 | 18.54 | 6.45 |
|  |  | Branquea | | | | *Pyrgulopsis manantiali* | | | | 5.08 | 1.57 | 3.23 |
|  |  |  |  |  | Coahuilix | | | | |  |  |  |
|  |  |  |  |  |  | Coahuilix hubssi | | | | 5.00 | 4.18 | 4.84 |
|  |  |  |  |  | *Mexipyrgus* | | | | |  |  |  |
|  |  |  |  |  |  | *Mexipyrgus carranzae* | | | | 2.21 | 6.79 | 4.84 |
| Chordata | | | | | | | | | |  |  |  |
|  | Actinopterygii | | | | | | | | |  |  |  |
|  |  | Teleostei | | |  |  |  |  |  |  |  |  |
|  |  |  | Cyprinodontiformes | | | | | | |  |  |  |
|  |  |  |  | Poeciliidae | | | | | |  |  |  |
|  |  |  |  |  | *Gambusia* | | | | |  |  |  |
|  |  |  |  |  |  | *Gambusia marshi* | | | | 2.87 | 9.66 | 4.84 |
|  |  |  | Vertebras | | | | | | | 0.25 | 0.52 | 1.61 |
| Nematoda | | | | | | | | | |  |  |  |
|  |  | Adenoforea | | | | | | | |  |  |  |
|  |  |  | Monhysterida | | | | | | |  |  |  |
|  |  |  |  | Siphonolaimidae | | | | | | 0.33 | 1.04 | 3.23 |
| Plantae | | | | | | | | | |  |  |  |
|  |  |  | Lamiales | | | | | | |  |  |  |
|  |  |  |  | Lentibulariaceae | | | | | |  |  |  |
|  |  |  |  |  | *Urticularia* | | | | |  |  |  |
|  |  |  |  |  |  | *Urticularia obtusa* | | | | 2.95 | 3.66 | 4.84 |
| Plantae Sp.2 | | | | | | | | | | 9.34 | 3.92 | 3.23 |
| Plantae Sp.3 | | | | | | | | | | 1.64 | 2.61 | 1.61 |
| Plantae Sp.4 | | | | | | | | | | 2.21 | 4.18 | 6.45 |
| Plantae Sp.5 | | | | | | | | | | 9.59 | 7.83 | 3.23 |
| Plantae Sp.7 | | | | | | | | | | 1.56 | 2.61 | 1.61 |
|  |  | Charophyceae | | | | | | | |  |  |  |
|  |  |  | Charales | | | | | | |  |  |  |
|  |  |  |  | Characeae | | | | | |  |  |  |
|  |  |  |  |  | *Chara* | | | | |  |  |  |
|  |  |  |  |  |  | *Chara* sp. | | | | 1.80 | 3.66 | 4.84 |
| **Total** | | | | | | | | | | **100.00** | **100.00** | **100.00** |

Table 9. Trophic spectrum of *Hemichromis guttatus*. Percent of number (% N), volumetric (% V), and frequency of occurrence (% FO).

| **Stomach contents** | | | | | | | | | | **%N** | **%V** | **%FO** |
| --- | --- | --- | --- | --- | --- | --- | --- | --- | --- | --- | --- | --- |
| Organic material | | | | | | | | | | 1.72 | 64.78 | 14.37 |
| Food items | | | | | | | | | | 98.28 | 35.22 | 85.63 |
| **Total** | | | | | | | | | | **100.00** | **100.00** | **100.00** |
| Food items | | | | | | | | | | **%N** | **%V** | **%FO** |
| Arthropoda | | | | | | | | | |  |  |  |
|  | Crustacea | | | | | | | | |  |  |  |
|  |  | Maxilopoda | | | | | | | |  |  |  |
|  |  |  | Copepoda 1 | | | | | | | 0.60 | 1.53 | 3.43 |
|  |  |  | Copepoda 2 | | | | | | | 4.61 | 1.90 | 3.71 |
|  |  | Malacostraca | | | | | | | |  |  |  |
|  |  |  | Amphipoda | | | | | | |  |  |  |
|  |  |  |  | Dogielinotidae | | | | | |  |  |  |
|  |  |  |  |  | *Hyalella* | | | | |  |  |  |
|  |  |  |  |  |  | *Hyalella azteca* | | | | 0.98 | 4.73 | 3.79 |
|  |  | Brachipoda | | | | | | | |  |  |  |
|  |  |  | Diplostraca | | | | | | |  |  |  |
|  |  |  |  | Chydoridae | | | | | | 5.37 | 2.22 | 4.14 |
|  |  |  | Cladocera | | | | | | | 0.24 | 0.62 | 2.00 |
|  |  | Ostracoda | | | | | | | |  |  |  |
|  |  |  | Podocopida | | | | | | |  |  |  |
|  |  |  |  | Cyprididae | | | | | |  |  |  |
|  |  |  |  |  | *Chlamydotheca* | | | | |  |  |  |
|  |  |  |  |  |  | *Chlamydotheca arcuata* | | | | 17.96 | 7.75 | 7.07 |
|  |  | Chelicerata | | | | | | | |  |  |  |
|  |  |  | Arachnida | | | | | | |  |  |  |
|  |  |  |  | Acari | | | | | |  |  |  |
|  |  |  |  |  | Trombidiformes | | | | |  |  |  |
|  |  |  |  |  |  | Prostigamata 1 | | | | 2.02 | 3.92 | 4.08 |
|  |  |  |  |  |  | Prostigamata 2 | | | | 0.20 | 0.50 | 1.50 |
|  |  |  |  |  | Acariformes 2 | | | | | 0.15 | 0.55 | 1.50 |
|  |  |  |  | Aranae 1 | | | | | | 0.03 | 0.11 | 0.43 |
|  |  |  |  | Aranae 2 | | | | | | 0.03 | 0.11 | 0.43 |
|  |  |  |  | Aranae 3 | | | | | | 0.05 | 0.14 | 0.21 |
|  |  |  |  | Aranae 4 | | | | | | 0.02 | 0.05 | 0.21 |
|  | Hexapoda | | | | | | | | |  |  |  |
|  |  | Insecta | | | | | | | |  |  |  |
|  |  |  | Ephemenoptera 2 | | | | | | | 0.12 | 0.90 | 0.04 |
|  |  |  |  | Leptohyphidae 1 | | | | | | 0.62 | 9.50 | 3.43 |
|  |  |  |  |  | *Tricorythodes* | | | | | 0.10 | 0.15 | 0.03 |
|  |  |  |  | Caenidae | | | | | |  |  |  |
|  |  |  |  |  | *Caenis* | | | | | 0.20 | 0.45 | 0.43 |
|  |  |  | Coleoptera | | | | | | |  |  |  |
|  |  |  |  | Belidae 1 | | | | | | 0.02 | 0.05 | 0.21 |
|  |  |  |  | Elmidae 1 | | | | | | 0.04 | 0.71 | 0.39 |
|  |  |  |  | Elmidae 2 | | | | | | 0.20 | 0.40 | 0.50 |
|  |  |  | Odonata | | | | | | |  |  |  |
|  |  |  |  | Anisoptera 1 | | | | | | 0.09 | 0.37 | 0.50 |
|  |  |  |  | Libellulidae 3 | | | | | | 0.02 | 0.06 | 0.32 |
|  |  |  | Diptera | | | | | | |  |  |  |
|  |  |  |  | Ceratopogonidae | | | | | |  |  |  |
|  |  |  |  |  | *Bezzia* | | | | |  |  |  |
|  |  |  |  |  |  | *Bezzias sp* | | | | 0.92 | 1.22 | 2.43 |
|  |  |  |  |  | *Culicoides* | | | | | 0.20 | 1.50 | 2.00 |
|  |  |  |  | Chironomidae 1 | | | | | | 19.00 | 11.00 | 9.00 |
|  |  |  |  | Chironomidae 2 | | | | | | 0.75 | 1.05 | 2.74 |
|  |  |  |  | Chironomidae 3 | | | | | | 1.01 | 0.98 | 1.29 |
|  |  |  |  | Chironomidae 5 | | | | | | 0.10 | 0.44 | 1.07 |
|  |  |  |  | Tipulidae 1 | | | | | | 0.29 | 1.93 | 1.73 |
|  |  |  |  | Tipulidae 4 | | | | | | 0.02 | 0.05 | 0.21 |
|  |  |  |  | Tipulidae 5 | | | | | | 0.02 | 0.05 | 0.20 |
|  |  |  |  | Tabanidae 1 | | | | | | 0.02 | 0.44 | 0.21 |
|  |  |  |  | Tabanidae 2 | | | | | | 0.02 | 0.38 | 0.21 |
|  |  |  |  | Tabanidae 3 | | | | | | 0.02 | 0.44 | 0.21 |
|  |  |  | Hymenoptera | | | | | | |  |  |  |
|  |  |  |  | Formacidae 1 | | | | | | 0.09 | 1.91 | 1.29 |
|  |  |  |  | Formacidae 2 | | | | | | 0.04 | 0.75 | 0.45 |
|  |  |  | Trichoptera | | | | | | |  |  |  |
|  |  |  |  | Leptoceridae 1 | | | | | | 0.02 | 0.16 | 0.21 |
|  |  |  | Hemiptera | | | | | | |  |  |  |
|  |  |  |  | Corixidae 1 | | | | | | 0.05 | 1.00 | 0.90 |
|  |  |  |  | Corixidae 2 | | | | | | 0.02 | 0.27 | 0.21 |
|  |  |  |  | Gerridae 1 | | | | | | 0.02 | 0.73 | 0.29 |
|  |  |  |  | Pleidae | | | | | |  |  |  |
|  |  |  |  |  | Neoplea | | | | | 0.02 | 0.16 | 0.21 |
|  |  |  |  |  | Nepidae | | | | | 0.02 | 0.16 | 0.21 |
|  |  | Insecta 2 | | | | | | | | 0.02 | 0.16 | 0.21 |
|  |  | Insecta 4 | | | | | | | | 0.04 | 0.05 | 1.20 |
|  |  | Insecta 10 | | | | | | | | 0.05 | 0.10 | 0.50 |
|  |  | Insecta 12 | | | | | | | | 0.03 | 0.33 | 0.21 |
|  |  | Insecta 13 | | | | | | | | 0.02 | 0.03 | 0.09 |
|  |  | Insecta 14 | | | | | | | | 0.02 | 0.16 | 0.21 |
|  |  | Insecta 15 | | | | | | | | 0.08 | 0.40 | 0.05 |
|  |  | Insecta 16 | | | | | | | | 0.06 | 0.03 | 0.03 |
|  |  | Insecta 17 | | | | | | | | 0.05 | 0.03 | 0.04 |
| Mollusca | | | | | | | | | |  |  |  |
|  |  | Gastropoda | | | | | | | |  |  |  |
|  |  |  | Mesogastropoda | | | | | | |  |  |  |
|  |  |  |  | Hydrobiidae 1 | | | | | | 0.12 | 0.93 | 0.90 |
|  |  |  |  | Hydrobiidae 2 | | | | | | 0.08 | 4.51 | 0.64 |
|  |  |  |  | Hydrobiidae 4 | | | | | | 0.02 | 0.16 | 0.21 |
|  |  |  |  |  | *Pyrgulopsis* | | | | |  |  |  |
|  |  |  |  |  |  | *Pyrgulopsis manantiali* | | | | 1.41 | 5.33 | 3.01 |
|  |  | Branquea | | | | *Pyrgulopsis manantiali* | | | | 0.13 | 0.36 | 0.79 |
|  |  |  |  |  | *Coahuilix* | | | | |  |  |  |
|  |  |  |  |  |  | *Coahuilix hubssi* | | | | 2.00 | 0.50 | 3.02 |
|  |  | Branquea | | | | *Coahuilix hubssi* | | | | 0.30 | 0.76 | 1.29 |
|  |  |  |  |  | *Mexipyrgus* | | | | |  |  |  |
|  |  |  |  |  |  | *Mexipyrgus carranzae* | | | | 0.05 | 0.27 | 0.21 |
|  |  |  |  |  | *Nymphophilus* | | | | |  |  |  |
|  |  |  |  |  |  | *Nymphophilus minckleyi* | | | | 0.80 | 0.87 | 0.43 |
| Chordata | | | | | | | | | |  |  |  |
|  | Actinopterygii | | | | | | | | |  |  |  |
|  |  | Teleostei | | |  |  |  |  |  |  |  |  |
|  |  |  | Cyprinodontiformes | | | | | |  |  |  |  |
|  |  |  |  | Poeciliidae | | | |  |  |  |  |  |
|  |  |  |  |  | Gambusia | | | |  |  |  |  |
|  |  |  |  |  |  | Gambusia marshi | | | | 0.03 | 1.09 | 0.21 |
|  |  |  | Radios | | | | | | | 0.18 | 0.44 | 0.64 |
|  |  |  | Branquiespinas | | | | | | | 0.02 | 0.22 | 0.21 |
|  |  |  | Escama cicloide | | | | | | | 1.69 | 2.08 | 4.00 |
|  |  |  | Escama ctenoide | | | | | | | 0.07 | 0.22 | 0.86 |
|  |  |  | Escamas de *H. guttatus* | | | | | | | 0.09 | 0.22 | 0.86 |
|  |  |  | Huevos | | | | | | | 0.03 | 0.05 | 0.21 |
| Nematoda | | |  |  |  |  |  |  |  |  |  |  |
|  |  | Adenoforea | | | |  |  |  |  |  |  |  |
|  |  |  | Monhysterida | | | |  |  |  |  |  |  |
|  |  |  |  | Siphonolaimidae | | | | |  | 0.03 | 0.08 | 0.36 |
| Plantae | | | | | | | | | |  |  |  |
|  |  |  | Lamiales | | | | | | |  |  |  |
|  |  |  |  | Lentibulariaceae | | | | | |  |  |  |
|  |  |  |  |  | *Urticularia* | | | | |  |  |  |
|  |  |  |  |  |  | *Urticularia obtusa* | | | | 3.12 | 5.24 | 5.31 |
| Plantae 1 | | | | | | | | | | 0.02 | 0.05 | 0.21 |
| Plantae 2 | | | | | | | | | | 0.47 | 1.75 | 2.00 |
| Plantae 4 | | | | | | | | | | 0.53 | 1.86 | 2.00 |
| Plantae 5 | | | | | | | | | | 0.08 | 2.29 | 0.21 |
| Plantae 7 | | | | | | | | | | 0.11 | 0.55 | 0.80 |
| Plantae 9 | | | | | | | | | | 30.40 | 0.38 | 0.60 |
| Plantae 11 | | | | | | | | | | 0.10 | 0.44 | 1.32 |
|  |  | Charophyceae | | | | | | | |  |  |  |
|  |  |  | Charales | | | | | | |  |  |  |
|  |  |  |  | Characeae | | | | | |  |  |  |
|  |  |  |  |  | *Chara* | | | | |  |  |  |
|  |  |  |  |  |  | *Chara* sp. | | | | 1.29 | 1.66 | 1.00 |
| N/I 1 | | | | | | | | | | 0.02 | 0.04 | 0.14 |
| N/I 2 | | | | | | | | | | 0.02 | 0.11 | 0.20 |
| N/I 3 | | | | | | | | | | 0.02 | 0.04 | 0.10 |
| N/I 6 | | | | | | | | | | 0.02 | 0.05 | 0.21 |
| N/I 7 | | | | | | | | | | 0.02 | 0.71 | 0.21 |
| N/I 8 | | | | | | | | | | 0.02 | 2.10 | 0.21 |
| N/I 12 | | | | | | | | | | 0.02 | 0.22 | 0.40 |
| N/I 13 | | | | | | | | | | 0.03 | 1.15 | 0.20 |
| N/I 14 | | | | | | | | | | 0.05 | 0.38 | 0.40 |
| N/I 15 | | | | | | | | | | 0.02 | 0.16 | 0.40 |
| N/I 16 | | | | | | | | | | 0.02 | 0.05 | 0.20 |
| **Total** | | | | | | | | | | **100.00** | **100.00** | **100.00** |

Table 9. Trophic spectrum of *Cyprinella xanthicara*. Percent of number (% N), volumetric (% V), and frequency of occurrence (% FO).

| **Stomach contents** | | | | | | | | | | **%N** | **%V** | **%FO** |
| --- | --- | --- | --- | --- | --- | --- | --- | --- | --- | --- | --- | --- |
| Organic material | | | | | | | | | | 4.79 | 31.93 | 21.13 |
| Food items | | | | | | | | | | 95.21 | 68.07 | 78.87 |
| **Total** | | | | | | | | | | **100.00** | **100.00** | **100.00** |
| Food items | | | | | | | | | | **%N** | **%V** | **%FO** |
| Arthropoda | | | | | | | | | |  |  |  |
|  | Crustacea | | | | | | | | |  |  |  |
|  |  | Maxilopoda | | | | | | | |  |  |  |
|  |  |  | Copepoda 1 | | | | | | | 3.69 | 1.67 | 3.57 |
|  |  |  | Copepoda 2 | | | | | | | 0.34 | 0.17 | 1.79 |
|  |  | Malacostraca | | | | | | | |  |  |  |
|  |  |  | Amphipoda | | | | | | |  |  |  |
|  |  |  |  | Dogielinotidae | | | | | |  |  |  |
|  |  |  |  |  | *Hyalella* | | | | |  |  |  |
|  |  |  |  |  |  | *Hyalella azteca* | | | | 16.11 | 47.25 | 16.07 |
|  |  | Brachipoda | | | | | | | |  |  |  |
|  |  |  | Cladocera | | | | | | | 10.74 | 3.01 | 12.50 |
|  |  | Ostracoda | | | | | | | |  |  |  |
|  |  |  | Podocopida | | | | | | |  |  |  |
|  |  |  |  | Cyprididae | | | | | |  |  |  |
|  |  |  |  |  | *Chlamydotheca* | | | | |  |  |  |
|  |  |  |  |  |  | *Chlamydotheca arcuata* | | | | 7.38 | 1.50 | 5.36 |
|  |  | Chelicerata | | | | | | | |  |  |  |
|  |  |  | Arachnida | | | | | | |  |  |  |
|  |  |  |  | Acari | | | | | |  |  |  |
|  |  |  |  |  | Trombidiformes | | | | |  |  |  |
|  |  |  |  |  |  | Hygrobatidea | | | | 1.34 | 0.67 | 3.57 |
|  | Hexapoda | | | | | | | | |  |  |  |
|  |  | Insecta | | | | | | | |  |  |  |
|  |  |  | Ephemenoptera | | | | | | |  |  |  |
|  |  |  |  | Baetidae 1 | | | | | | 0.67 | 2.50 | 1.79 |
|  |  |  |  | Baetidae 2 | | | | | | 1.34 | 6.68 | 3.57 |
|  |  |  | Odonata 2 | | | | | | | 0.34 | 0.50 | 1.79 |
|  |  |  | Diptera | | | | | | |  |  |  |
|  |  |  |  |  | *Culicoides* | | | | | 3.02 | 2.17 | 8.93 |
|  |  |  |  | Chironomidae 1 | | | | | | 3.69 | 2.17 | 10.71 |
|  |  |  |  | Chironomidae 2 | | | | | | 45.97 | 27.55 | 17.86 |
|  |  |  |  | Chironomidae 3 | | | | | | 0.34 | 0.17 | 1.79 |
|  |  | Insecta 1 | | | | | | | | 3.36 | 2.50 | 1.79 |
| Mollusca | | | | | | | | | |  |  |  |
|  |  | Gastropoda | | | | | | | |  |  |  |
|  |  |  | Mesogastropoda | | | | | | |  |  |  |
|  |  |  |  | Hydrobiidae 1 | | | | | | 0.34 | 0.33 | 1.79 |
| Chordata | | | | | | | | | |  |  |  |
|  | Actinopterygii | | | | | | | | |  |  |  |
|  |  | Teleostei | | |  |  |  |  |  |  |  |  |
|  |  |  | Escamas cicloides | | | | | | | 0.34 | 0.17 | 1.79 |
| Plantae 7 | | | | | | | | | | 0.34 | 0.50 | 1.79 |
| N/I 10 | | | | | | | | | | 0.34 | 0.33 | 1.79 |
| N/I 17 | | | | | | | | | | 0.34 | 0.17 | 1.79 |
| **Total** | | | | | | | | | | **100.00** | **100.00** | **100.00** |
