# Supplementary figures and images for: Trophic analysis of the fish community in the Ciénega Churince, Cuatro Ciénegas, Coahuila
Source: PeerJ. 2017 Sep 4;5:e3637. doi: 10.7717/peerj.3637 (PMC5588786; doi:10.7717/peerj.3637)

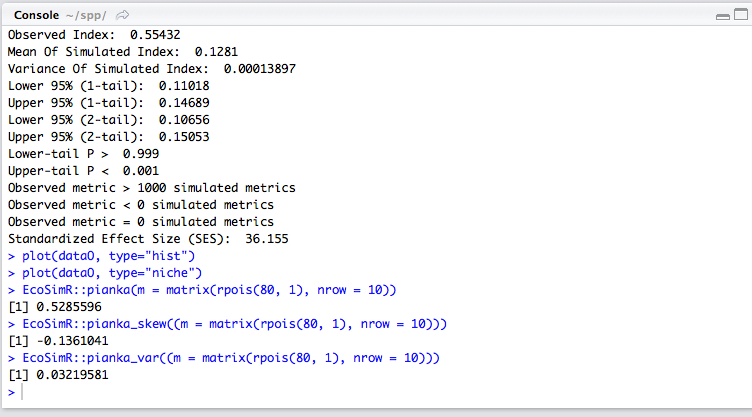

Supplement: Supplemental Information 4 [file peerj-05-3637-s004.png]
